# The Genomic Landscape, Causes, and Consequences of Extensive Phylogenomic Discordance in Murine Rodents

**DOI:** 10.1093/gbe/evaf017

**Published:** 2025-02-04

**Authors:** Gregg W C Thomas, Jonathan J Hughes, Tomohiro Kumon, Jacob S Berv, C Erik Nordgren, Michael Lampson, Mia Levine, Jeremy B Searle, Jeffrey M Good

**Affiliations:** Division of Biological Sciences, University of Montana, Missoula, MT 59801, USA; Informatics Group, Harvard University, Cambridge, MA 02138, USA; Department of Ecology and Evolutionary Biology, Cornell University, Ithaca, NY 14853, USA; Department of Evolution, Ecology, and Organismal Biology, University of California Riverside, Riverside, CA 92521, USA; Department of Biology, University of Pennsylvania, Philadelphia, PA 19104, USA; Department of Ecology and Evolutionary Biology, Cornell University, Ithaca, NY 14853, USA; Department of Ecology and Evolutionary Biology, University of Michigan, Ann Arbor, MI 48109, USA; Department of Biology, University of Pennsylvania, Philadelphia, PA 19104, USA; Department of Biology, University of Pennsylvania, Philadelphia, PA 19104, USA; Department of Biology, University of Pennsylvania, Philadelphia, PA 19104, USA; Department of Ecology and Evolutionary Biology, Cornell University, Ithaca, NY 14853, USA; Division of Biological Sciences, University of Montana, Missoula, MT 59801, USA

**Keywords:** phylogenetic discordance, murine rodents, molecular evolution recombination, genomics, mouse

## Abstract

A species tree is a central concept in evolutionary biology whereby a single branching phylogeny reflects relationships among species. However, the phylogenies of different genomic regions often differ from the species tree. Although tree discordance is widespread in phylogenomic studies, we still lack a clear understanding of how variation in phylogenetic patterns is shaped by genome biology or the extent to which discordance may compromise comparative studies. We characterized patterns of phylogenomic discordance across the murine rodents—a large and ecologically diverse group that gave rise to the laboratory mouse and rat model systems. Combining recently published linked-read genome assemblies for seven murine species with other available rodent genomes, we first used ultraconserved elements (UCEs) to infer a robust time-calibrated species tree. We then used whole genomes to examine finer-scale patterns of discordance across ∼12 million years of divergence. We found that proximate chromosomal regions tended to have more similar phylogenetic histories. There was no clear relationship between local tree similarity and recombination rates in house mice, but we did observe a correlation between recombination rates and average similarity to the species tree. We also detected a strong influence of linked selection whereby purifying selection at UCEs led to appreciably less discordance. Finally, we show that assuming a single species tree can result in substantial deviation from the results with gene trees when testing for positive selection under different models. Collectively, our results highlight the complex relationship between phylogenetic inference and genome biology and underscore how failure to account for this complexity can mislead comparative genomic studies.

**Significance**

Genomic data have demonstrated that when sequences from multiple species are compared, different regions of the genome exhibit different phylogenetic histories. These discordant histories could be due to either biological processes, such as ancestral variation or introgression, or artifacts of the inference process. We use the genomes of several murine rodents to distinguish how features of the genome, such as recombination rates, genes, and other conserved regions, affect this discordance across the genome. Considering the prevalence of discordance across the genome, we also test how using a single species tree, a common practice, affects inferences from tests for positive selection. Our study shows that conserved genomic loci exhibit lower amounts of discordance, and that discordance can negatively affect inferences of selection.

## Introduction

Phylogenies are a unifying concept in understanding the evolution of species, traits, and genes. However, extensive high-throughput sequencing data has now revealed that evolutionary relationships between species are often not be well represented by a single phylogeny (Edwards 2009; Hahn and Nakhleh 2016). While a dominant signal of bifurcating speciation usually exists (i.e. a species tree), phylogenetic signal that may disagree with species relationships can arise from ancestral polymorphisms (incomplete lineage sorting [ILS]), gene flow (introgression), and gene duplication and loss (Maddison 1997). The theoretical prediction of phylogenetic discordance has long been appreciated (Hudson 1983; Pamilo and Nei 1988; Maddison 1997; Rosenberg 2002), but empirical evidence now emphasizes just how extensive discordance can be among a set of species (Feng et al. 2022; Gable et al. 2022; Smith et al. 2023). For example, studies of birds (Jarvis et al. 2014), mammals (Ferreira et al. 2021; Lopes et al. 2021; Foley et al. 2024), plants (Pease et al. 2016), and insects (Sun et al. 2021; He et al. 2023) have found that with extensive taxon sampling and genomic data, highly supported species tree topologies are rarely or never exactly recovered in the underlying gene trees. Whereas these examples highlight the prevalence of phylogenetic discordance across the tree of life, we still lack a clear understanding of how phylogenetic patterns are shaped by the details of genome biology or the extent to which discordance may compromise inferences from comparative studies that assume a singular species history.

In practice, failure to acknowledge and account for phylogenetic discordance could severely affect biological inference. Analyses of molecular evolution are usually performed on a gene-by-gene basis (Pond et al. 2005; Yang 2007; Hu et al. 2019; Kowalczyk et al. 2019), but it is still common practice to assume a single genome-wide species tree for each locus. For gene-based analyses, using the wrong tree may cause erroneous inferences of positive directional selection, convergent evolution, and genome-wide inferences of correlated rate variation (Mendes et al. 2016). Phylogenetic discordance can also affect how continuous traits are reconstructed across phylogenies, as the genes that underly these traits may not follow the species history (Avise and Robinson 2008; Hahn and Nakhleh 2016; Mendes et al. 2018; Hibbins et al. 2023). In these instances, phylogenetic discordance may need to be characterized and incorporated into the experimental and analytical design. Alternatively, if a researcher's primary questions are focused on reconstructing the evolutionary history of speciation (i.e. the species tree), then phylogenetic discordance may obscure the true signal of speciation (Fontaine et al. 2015; Foley et al. 2024). In this case, knowledge about patterns of discordance across genomes could inform decisions about locus selection, data filtering, and model parameters during species tree reconstruction.

Given these considerations, a better understanding of the genomic context of phylogenetic discordance is warranted. Although often conceptualized primarily as a stochastic consequence of population history (Maddison 1997), patterns of phylogenetic discordance are likely to be nonrandom and dependent on localized patterns of genetic drift, natural selection, recombination, and mutation (Johri et al. 2022). Discordance due to ILS ultimately depends on effective population sizes across the branches of the phylogeny (Pamilo and Nei 1988; Degnan and Rosenberg 2006) and, therefore, should covary with any process that influences local patterns of genetic diversity (e.g. linked negative or positive selection). Likewise, discordance due to introgression may be influenced by selection against incompatible alleles or positive selection for beneficial variants (Lewontin and Birch 1966; Jones et al. 2018). Selection, ILS, and introgression, are expected to leave different genomic signals that should allow us to test hypotheses about both the cause and the scale of phylogenetic discordance (Huson et al. 2005; Kulathinal et al. 2009; Green et al. 2010; Vanderpool et al. 2020). Yet, the genomic context of phylogenetic discordance has remained elusive. For example, localized patterns of phylogenetic discordance should be influenced by patterns of recombination (Hudson and Kaplan 1988) and simulation studies confirm that the closer two regions are in the genome, the more history they share (Slatkin and Pollack 2006; McKenzie and Eaton 2020). However, empirical studies have been inconclusive regarding the relationship between discordance and recombination rates, ranging from no relationship in great apes (Hobolth et al. 2007), a weak positive correlation in house mice (White et al. 2009), a strong positive correlation broadly across primates (Rivas-Gonzalez et al. (2023), or increased discordance in regions of lower recombination (Scally et al. 2012; Pease and Hahn 2013). Thus, it remains unclear how phylogenetic discordance scales locally across the genome as a function of recombination and the strength of linked selection, pointing to the need for empirical studies in systems with sufficient genomic resources to explore the causes of discordance.

To investigate the causes and consequences of phylogenetic discordance, we took advantage of genomic resources available for house mice (*Mus musculus*). This rodent species is one of the most important mammalian model systems for biological and biomedical research and is embedded within a massive radiation of rats and mice (Murinae). This ecologically diverse and species-rich group is comprised of over 600 species and makes up >10% of all mammalian species, and yet is only about 15 million years old, making this system an excellent choice for phylogenetic studies over both short and long timescales. Despite the power of evolution-guided functional and biomedical analysis (Christmas et al. 2023), relatively few murine genomes have been sequenced outside of *Mus* and *Rattus*.

We analyze recently sequenced genomes for seven murine species (*Mastomys natalensis*, *Hylomyscus alleni*, *Praomys delectorum*, *Rhabdomys dilectus*, *Grammoyms dolichurus*, *Otomoys typus*, and *Rhynchomys soricoides*) sampled from across this radiation (Kumon et al. 2021). We combine these new genomes with previously sequenced genomes and genomic resources from the *M. musculus* model system to study phylogenetic relationships within Murinae as well as the landscape of discordance along rodent chromosomes. We first inferred a species tree for these and other sequenced rodent genomes, focusing on signals derived from commonly used ultraconserved elements (UCEs). We used these UCE data to infer a robust, time-calibrated phylogeny of sequenced murine rodents, providing a useful resource for future comparative studies within this important group. Using this species tree, we then used a subset of whole genomes to study how phylogenetic discordance is related to species-level inferences of relatedness, recombination rate, and patterns of molecular evolution. Using genetic maps and functional annotation from the powerful house mouse system, we test several hypotheses linking spatial patterns of discordance to genetic drift, natural selection, and recombination. Finally, we show how the use of a single species tree impacts gene-level inferences from common molecular evolution tests for natural selection in these species. Collectively, our results advance our understanding of how core features of genome biology influence underlying phylogenetic patterns, the extent to which established model system resources can be leveraged for broader phylogenetic studies, and the consequences of ignoring phylogenetic uncertainty.

## Results

### Estimation of a Murine Species Tree

**Fig. 1.**
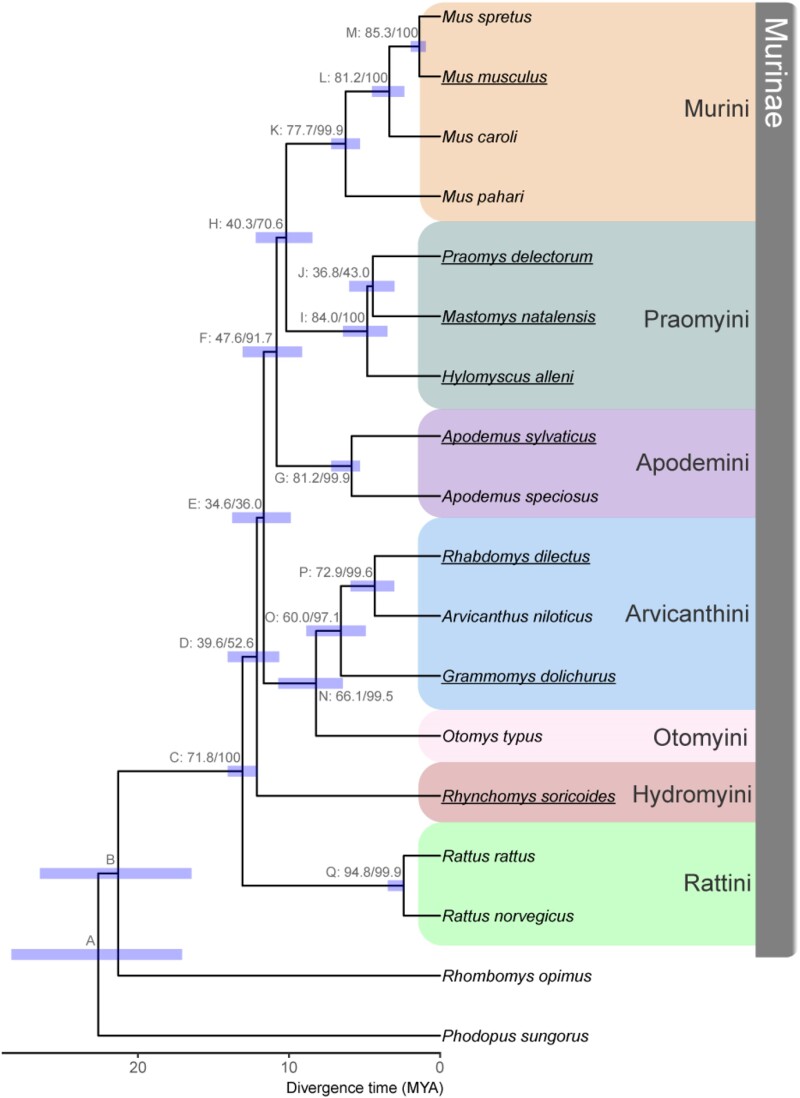
Species trees inferred from concatenation of UCEs from 18 rodent species. Internal nodes are labeled by a letter identifier referenced in the text and site and gene concordance factors (i.e. label: sCF/gCF) as well as a bar indicating the CI for divergence time estimation. Ultrafast bootstrap/SH-aLRT values were all 100. Bottom scale represents time in millions of years before present. Fossil calibrations are described in supplementary tables S2 and S3, Supplementary Material online. Tribes within subfamily Murinae are highlighted on the right following the classifications used by Lecompte et al. (2008). Genomes used for the genome-wide phylogenetic discordance analyses are underlined.

Using a concatenated dataset of 2,632 aligned UCEs, we inferred a species tree of 18 murine rodent species (Fig. 1; supplementary table S1, Supplementary Material online) that recovered the same relationships as previous reconstructions of Murinae using a small number of loci (Lecompte et al. 2008; Steppan and Schenk 2017). The species tree inferred from a quartet-based summary of the gene tree topologies was identical to the concatenated tree (supplementary fig. S1, Supplementary Material online). While bootstrap and SH-aLRT values provided high support to our inferred species trees (Fig. 1), we found evidence for considerable discordance across individual UCE phylogenies. The five shortest branches in the concatenated tree had a site concordance factor (sCF) of less than 50%, suggesting that alternate resolutions of the quartet had equivocal support (supplementary fig. S2, Supplementary Material online). Gene concordance factors (gCF) for each branch in the species tree were on aggregate much higher, with all but four branches supported by almost every gene tree in the analysis and with the lowest values likely being driven by a several short internal branches (supplementary fig. S2, Supplementary Material online). This pattern was recapitulated using a quartet-based summary method (supplementary figs. S1 and S3, Supplementary Material online). At the two most discordant nodes (E and J in Fig. 1), the recovered topology was supported by approximately one-third of all gene trees.

We estimated divergence times for the inferred concatenated phylogeny (Fig. 1; supplementary table S2, Supplementary Material online) using four fossil calibration points (supplementary table S3, Supplementary Material online). The murid and cricetid groups had an estimated divergence time of 22.66 Ma (node A in Fig. 1) followed by the Murinae and the Gerbillinae at 21.34 Ma (B), albeit with wide confidence intervals (CI) in both cases. The core Murinae (C) sensu Steppan et al. (2005) is inferred to have arisen 13.11 Ma (CI: 11.42–15.10). Hydromyini then split off at 12.15 Ma (D, CI: 11.10–13.51) followed by Otomyini and Arvicanthini at 11.70 Ma (E, fossil calibration from Kimura et al. 2015). The remaining Murine tribes evolved in rapid succession, with Apodemini diverging from Murini and Praomyini at 10.84 Ma (F). Murini and Praomyini then split at 10.19 Ma (H). The two *Rattus* species in our dataset were inferred to have diverged 2.01 Ma (Q, CI: 1.26–2.30). This dated UCE phylogeny is congruent with previous works (Lecompte et al. 2008; Steppan and Schenk 2017) and provides context on the evolutionary timescale upon which we next describe the genomic landscape of phylogenetic discordance across a collection of murine genomes.

### The Landscape of Phylogenetic Discordance Along Murine Genomes

We analyzed genome-wide phylogenetic histories of six recently sequenced murine rodent genomes and the *M. musculus* reference genome spanning approximately 12 million years of divergence (see Fig. 1). Using the *M. musculus* coordinate system, we partitioned and aligned 263,389 nonoverlapping 10 kb windows from these seven species (supplementary table S1, Supplementary Material online). After filtering windows in repetitive regions or with low phylogenetic signal, we recovered 163,765 trees with an average of 616 informative sites per window (supplementary fig. S4, Supplementary Material online).

**Fig. 2.**
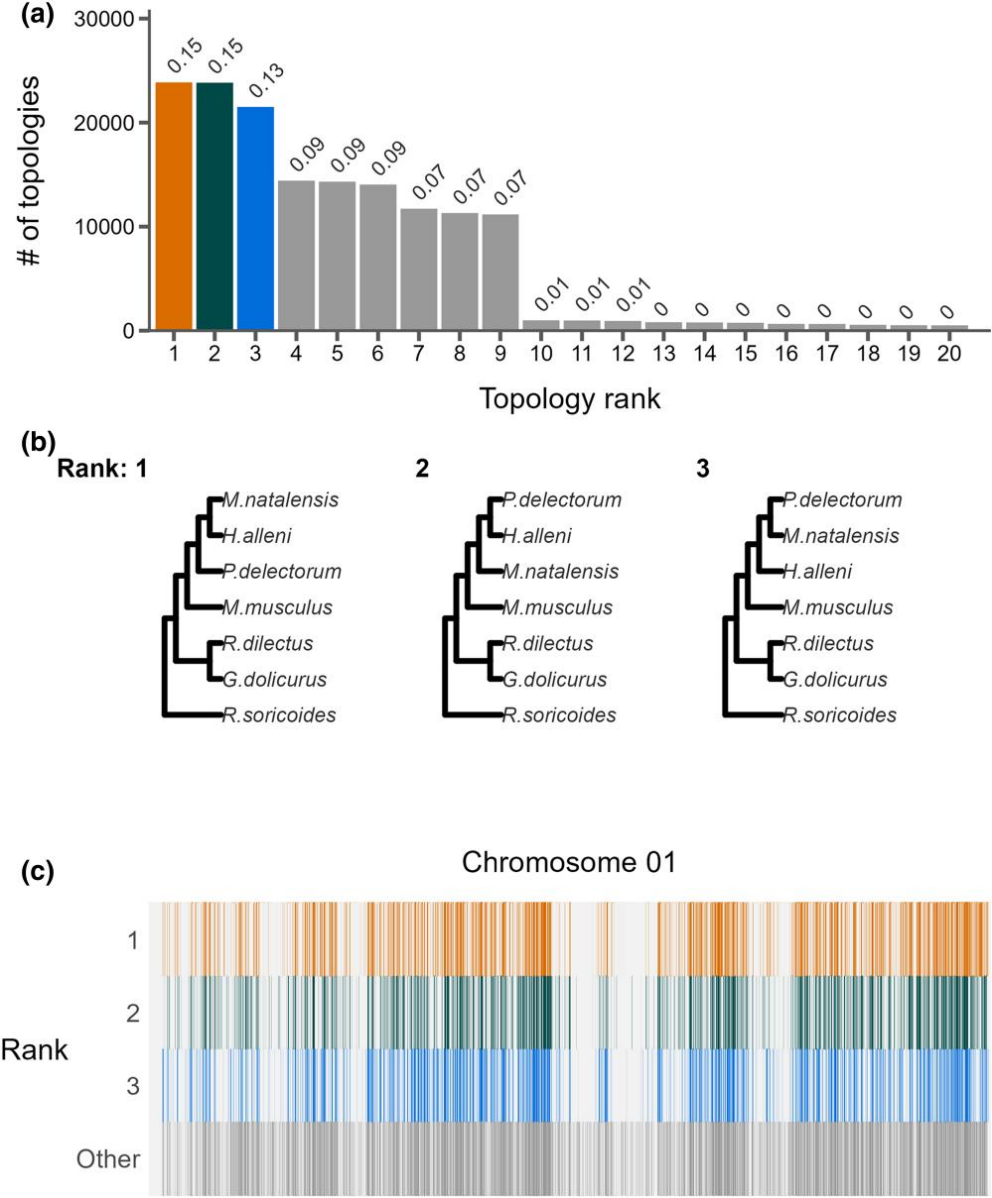
The landscape and profile of phylogenetic discordance across nonoverlapping 10 kb windows in murine genomes. a) Distribution of the 20 most frequent topologies recovered across all windows. Numbers above bars indicate proportion of each topology. b) The top three topologies recovered across all chromosomes 1. c) Distribution of the topologies recovered along chromosome 1. The *x* axis is scaled to the length of the chromosome and each vertical bar represents one 10 kb window. The three most frequent topologies occupy the first three rows while all other topologies are shown in the bottom row. See supplementary File S1, Supplementary Material online for individual chromosome plots.

Phylogenetic discordance was pervasive within and between chromosomes. We inferred 597 of the 945 possible unique rooted topologies among six species (when specifying *R. soricoides* as the outgroup) across all chromosomes. The number of unique topologies per chromosome ranged from 75 to 218 (mean = 141). However, just four different topologies were ranked in the top three per chromosome. (Fig. 2a; supplementary file S1, Supplementary Material online) and only nine trees were present at a frequency above 1%. Among these, the top three topologies only differed in the ordering of the clade containing *H. alleni*, *M. natalensis*, and *P. delectorum* (HMP clade). This clade also showed the second lowest concordance in the species tree inferred from UCEs (Fig. 1, node J). These three topologies comprise between 13% and 15% of all recovered topologies (Fig. 2). Interestingly, the least common of these three trees (13.1%) matched the topology recovered via concatenation of all coding regions and the species tree recovered from UCEs (Fig. 1). That is, the robustly inferred species tree did not match the evolutionary relationships inferred for over 85% of the genome.

**Fig. 3.**
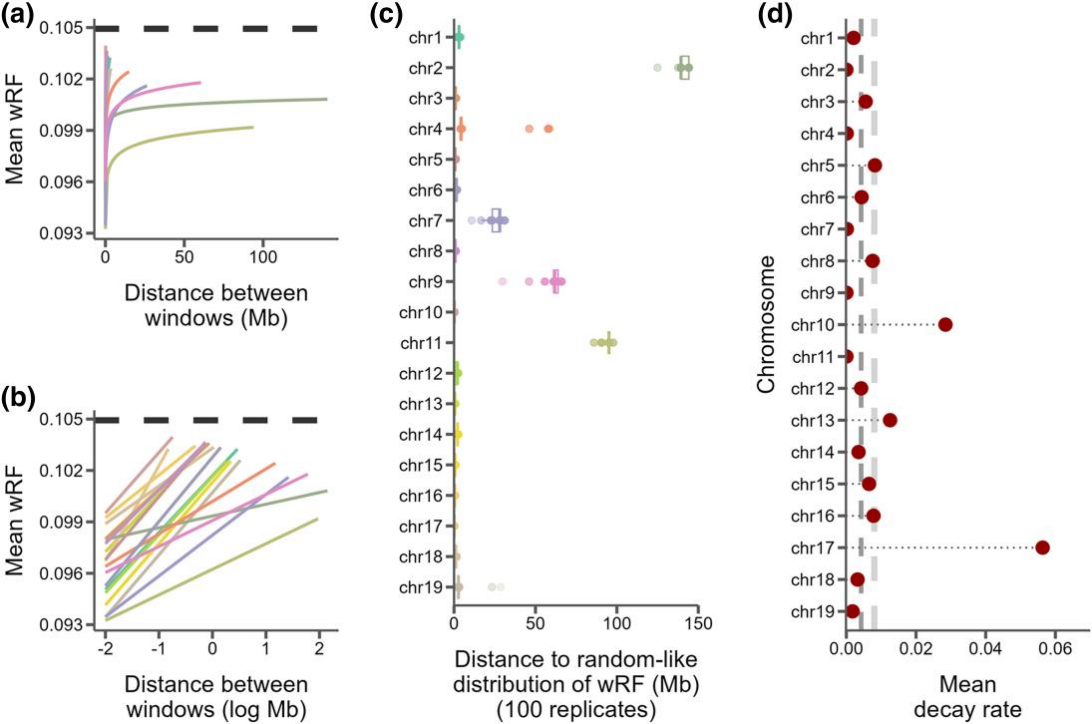
Similarity between 10 kb windows decays as genomic distance between windows increases. a) The log fit to the mean of distributions of wRF distances between trees of windows at increasing genomic distance (10 kb steps). Each line represents one chromosome. b) The same, but on a log scale with a linear fit. c) For every window on each chromosome, the genomic distance between windows at which tree distance becomes random for 100 replicates of random window selection. d) Points represent the slopes of the correlation between genomic distance and tree distance (lines from b), which is the rate at which tree similarity decays across the genome. Dark grey dashed line is median slope and light grey dashed line is mean.

While visual inspection revealed no clear partitioning of topological structures along chromosomes (e.g. Fig. 2c), we found that phylogenies were not randomly distributed across mouse chromosomes. Using the weighted Robinson–Foulds (wRF) metric, we found that tree similarity between windows decayed logarithmically along chromosomes (Fig. 3a and b), and the distance at which tree similarity appeared random varied considerably among chromosomes ranging from 0.15 Megabases (Mb) on chromosome 17 to 141.29 Mb on the chromosome 2 (Fig. 3c, supplementary fig. S5, Supplementary Material online). While chromosomes 2, 7, 9, and 11 were autosomal outliers with distances between windows to random-like trees exceeding 25 Mb, the average distance among all other autosomes was only 2.1 Mb. The rates at which phylogenetic similarity decayed tended to be inversely proportional to the distance at which two randomly drawn phylogenies lost similarity (Fig. 3d).

Next, we performed a pairwise alignment of the reference mouse and rat genomes to assess how large structural variations, such as inversions and translocations, may influence our inferences of phylogenetic relatedness along the genome. These species span the deepest divergence of the sample for which we assessed genome-wide discordance, so the level of large structural variation present among them should give us an idea of the amount of ancestral variation in our sample. The mouse and rat genomes were mostly colinear for large, aligned chunks, with large translocations and inversions on mouse chromosomes 5, 8, 10, 13, and 16 (supplementary fig. S6, Supplementary Material online). We also observe large-scale inversions on chromosome 16. We found that, while most chromosomes were colinear between mouse and rat, the average size of the 307,275 contiguously aligned chunks averages under 10 kb, with the average distance between aligned segments being between 2,380 bp on the mouse genome and 4,927 bp on the rat chromosome (supplementary fig. S7, Supplementary Material online). This pattern presents two major implications for our analyses. First, we could not transpose the coordinate system from mouse to rat with enough resolution to use genetic maps from rat. Second, most other structural variations in our sample appear likely to be small insertions of transposable elements (e.g. SINEs ∼150–500 bp, LINEs ∼4–7 kb; Platt et al. 2018) that should have a negligible effect on discordance analyses since our window size is much larger, and we excluded windows that were made up of mostly repeats.

### Discordance With Recombination Rate and Other Genomic Features

**Fig. 4.**
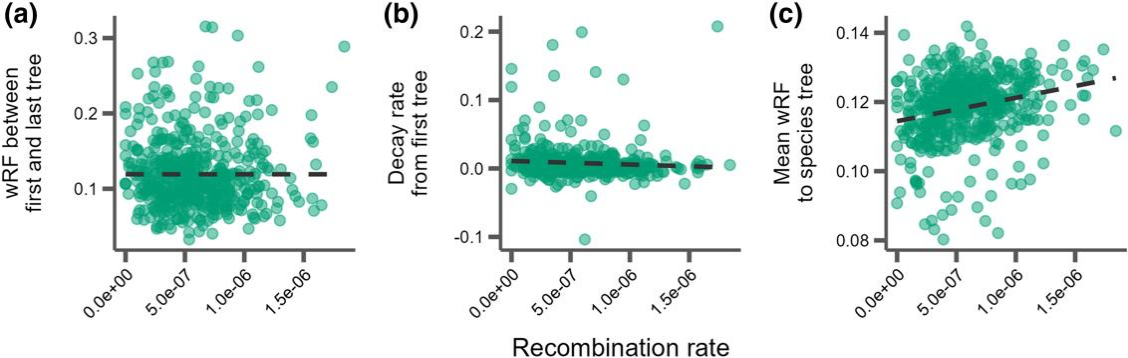
Correlations between tree similarity and recombination rate in 5 Mb windows. a) Tree similarity as measured by the wRF distance between the first and last 10 kb windows within the 5 Mb window. b) The slopes of the linear correlation between the wRF distances between the first 10 kb window and every other 10 kb window within a 5 Mb window represent the rate at which tree similarity decays over each 5 Mb window. c) The mean wRF of all 10 kb window trees within each 5 Mb window compared to the species tree.

**Fig. 5.**
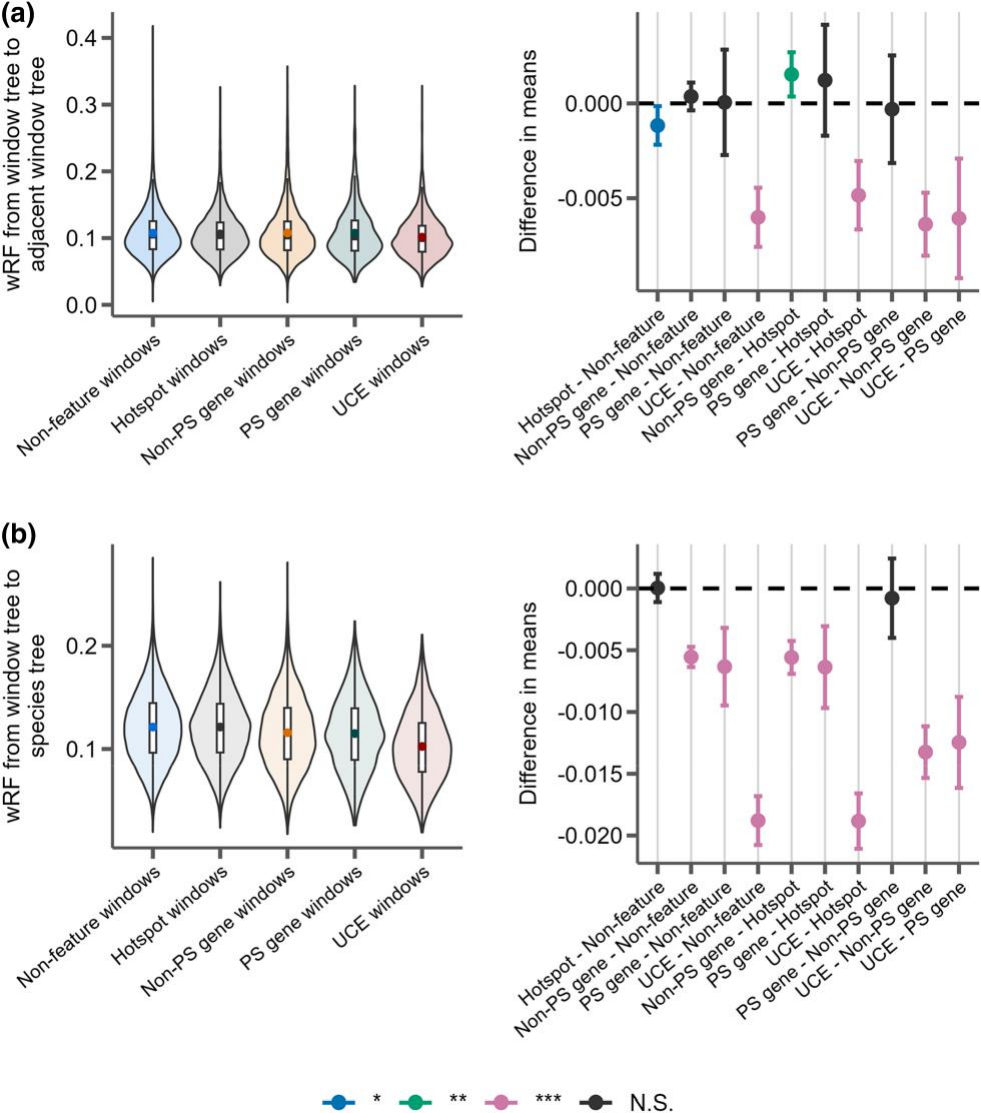
Distributions of wRF distance from trees constructed from 10 kb windows either centered on recombination hotspots (Hotspot), protein-coding genes without evidence for positive selection (Non-PS genes), protein-coding genes with evidence for positive selection (PS genes), UCEs, or containing none of these features (Nonfeature). For each panel, the left portion shows the distributions of the measure for each feature type and the right panel shows the differences in means for each pairwise comparison of features with significance assessed with Tukey's range test. The labels on the *x* axis indicate the feature pairs being compared, with the first feature being the reference (i.e. points above 0 indicate this feature has a higher mean). *P-*value thresholds: * < 0.05, ** < 0.01, *** < 0.001. a) The phylogenetic similarity of windows immediately adjacent to feature windows. b) The phylogenetic similarity between the species tree inferred from protein-coding gene trees and the feature window.

Using markers from genetic crosses within *M. musculus* (Shifman et al. 2006; Cox et al. 2009), we examined whether regions with high recombination also showed more phylogenetic discordance over short genetic distances when compared to regions with low recombination. Specifically, we calculated recombination rates within 5 Mb windows (supplementary fig. S8, Supplementary Material online) and then measured tree similarity between the first and last 10 kb window (*R*^2^ = 3.0e−9; *P* = 0.99; Fig. 4a) and the rate at which tree similarity changes between the first 10 kb window and every other 10 kb window (*R*^2^ = 0.003; *P* = 0.11; Fig. 4b). Surprisingly, we found no relationship between tree similarity and recombination rates measured at this scale. However, we did observe a slight positive correlation between recombination rate and dissimilarity to the species tree when averaging wRF over all 10 kb window trees within a 5 Mb recombination window (*R*^2^ = 0.05; *P* = 7.6e−8; Fig. 4c). We also examined regions of the genome centered on recombination hotspots identified in *M. musculus* (Smagulova et al. 2011) and found that these regions had significantly slower rates of decay in similarity over genomic distance compared to windows that were not centered on hotspots (*P* = 0.019; Fig. 5a), and that they were also significantly more phylogenetically similar over short distances (*P* = 0.015; Fig. 5b). Thus, when taken as a whole, we found that regions of higher recombination rates in house mice did not show more local phylogenetic discordance per se but did tend to show more discordance relative to the genome-wide species tree.

Evolutionary relationships around certain conserved genomic features may also be shaped by locally reduced effective population sizes due to a history of pervasive linked negative or positive selection. To test for this, we measured tree similarity in 10 kb windows around all annotated protein-coding genes, UCEs, and protein-coding genes identified as evolving rapidly (i.e. significantly elevated *d_N_*/*d_S_*) due to positive directional selection and compared these patterns relative to chromosome-wide trends (i.e. windows without annotated features). In general, UCEs showed more local phylogenetic similarity among adjacent windows (i.e. less discordance) than regions surrounding recombination hotspots (*P* = 2.42e−12), coding genes (*P* = 4.65e−14), rapidly evolving coding genes (*P* = 1.56e−6), and windows that did not include any of these features (*P* = 5.02e−14; Fig. 5a). In contrast, protein-coding genes (including rapidly evolving genes) were indistinguishable from background rates of discordance observed in windows without annotated genomic features (Fig. 5a). Likewise, UCEs were also much more similar to the overall species tree when compared to any other feature (Fig. 5b). Unlike our test of local discordance, protein-coding genes also showed less species tree discordance than windows containing no features or recombination hotspots, but the effect was much less pronounced than observed at UCEs.

### Consequences of Tree Specification on Analyses of Molecular Evolution

Next, we examined how phylogenetic discordance influenced inferences on the evolution of protein-coding sequences. Among a set of 22,261 *M. musculus* protein-coding transcripts, the average distance between the start and end of the coding sequence was 37.02 kb, or roughly four nonoverlapping 10 kb windows. At this distance, tree similarity is predicted to diminish considerably (e.g. by 0.10 wRF units), such that the phylogenetic history of individual genes may often contain some phylogenetic discordance (Mendes and Hahn 2016; Mendes et al. 2019). We also found that out of the 67,566 times the coding sequence in a gene overlapped with a 10 kb window, the inferred topology of the gene tree exactly matched the topology of the corresponding window tree only 11% of the time. Thus, the common practice of inferring gene trees on concatenated coding exons from a single transcript is still likely averaging over multiple possible albeit correlated histories.

**Fig. 6.**
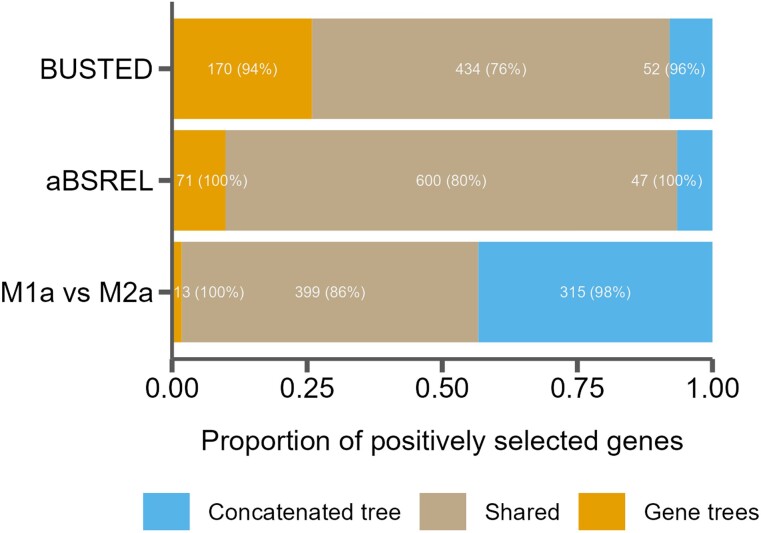
The proportion of genes inferred to be under positive selection for three tests using either a single species tree (concatenated tree) or individual gene trees, as well as those found in both cases (shared). Numbers in the bars indicate raw counts, and percentages indicate the percent of genes in that category that are discordant from the species tree.

Finally, we tested how tree misspecification might impact standard *d_N_*/*d_S_* based phylogenetic analyses for positive directional selection. Specifically, we used the still common practice of assuming a single species tree for all genes and compared that to using individually inferred gene trees in three common statistical tests for positive selection: PAML's M1a vs. M2a test (Yang 2007), HyPhy's BUSTED test (Murrell et al. 2015), and HyPhy's aBSREL test (Smith, Wertheim, et al. 2015). We found that many genes were inferred as having experienced positive directional selection when using a single species tree, but not when using gene trees and vice versa (Fig. 6). The extent to which the single species tree differed from the gene trees for the different types of selection test is documented in Table 1. For BUSTED, we observe that 28% of genes inferred as having evolved under positive directional selection when using the gene tree were not inferred when using the concatenated species tree. The opposite was true for M1a vs. M2a, where, among genes showing inconsistent evidence for positive selection across the two scenarios, 76% do so when using the concatenated species tree but not individual gene trees. In general, genes found to be evolving under positive selection using both tree types tended to be more concordant with the species tree than those that had evidence for positive selection either using only the concatenated tree or the gene tree (Fig. 6).

**Table 1:** Incidence where a single species tree does not match the gene tree expectation in three different tests for positive selection, either not detecting positive selection when it is inferred using the gene tree (undetected selection) or by detecting positive selection that is not inferred when using the gene tree (newly detected selection)

Test	Undetected selection (%)	Newly detected selection (%)
BUSTED	0.45	28.10
aBSREL	0.41	10.60
M1a vs. M2a	2.66	3.20

## Discussion

Phylogenies provide insight into the relationships of species and serve as a framework for asking questions about molecular and trait evolution. However, phylogenetic histories vary extensively across regions of a genome as a simple consequence of population genetic processes, and evolutionary relationships between species may not often be well represented by a single representative species-level phylogeny. Here, we combine the resources of the house mouse (*M. musculus*) with new and recently published (Kumon et al. 2021) genomes from seven species to understand the systematics of murine rodents and causes and consequences of phylogenetic discordance along murine genomes. These new analyses help to place this important model system in a stronger evolutionary context and begin to fill the gap in genome sampling of murine rodents, which, despite their exceptional morphological and ecological diversity and species richness, have had relatively few whole genomes sequenced. We use these genomic resources to study the landscape of phylogenetic discordance across the genome, understand how recombination and natural selection shape phylogenetic histories, and evaluate how assuming a single evolutionary history can compromise the study of molecular evolution in an important biomedical model system.

### Phylogenomic Relationships of Murine Rodent Lineages from Conserved Genomic Regions

The extraordinary species richness of murine rodents complicates phylogenetic analyses because of the resources required to sample, sequence, and analyze such widely distributed taxa. Earlier work either attempted to resolve specific groups such as *Mus* (Lundrigan et al. 2002; Suzuki et al. 2004) and *Apodemus* (Serizawa et al. 2000; Liu et al. 2004), or to uncover broader relationships across the subfamily (Martin et al. 2000; Steppan et al. 2005) based on a few genetic markers. Lecompte et al. (2008) provided one of the earliest well-supported phylogenetic reconstructions from across Murinae and the tribal classifications they proposed remain generally supported. More recent work has increased the number of taxa sampled, both for analyses of Murinae specifically (Pagès et al. 2016) and for their placement within Muridae and Muroidea (Schenk et al. 2013; Steppan and Schenk 2017; Rowe et al. 2019) albeit based on a limited number of loci. More recent studies have greatly expanded the number of loci used for phylogenetic inference (Mikula et al. 2021), including the use of 1,245 exons (Roycroft et al. 2020) and 1,360 exons (Roycroft et al. 2021), but have focused on specific tribes within Murinae.

Our inferred species tree based on 2,632 UCEs from 18 species across the radiation (Fig. 1) is consistent with previous studies (Lecompte et al. 2008; Steppan and Schenk 2017; Aghova et al. 2018). Branch support was uniformly high, and gene trees unambiguously support the tribal classification of Lecompte et al. (2008). However, four shorter branches show more substantial gene tree discordance (Fig. 1, branches D, E, H, and J), with two recovered clades (E and J) being supported by less than half of all gene trees. We also estimated divergence times on our inferred species tree using four fossil calibration points (supplementary table S3, Supplementary Material online), recovering times that are roughly consistent with the relatively young estimates found by Steppan and Schenk (2017) (see supplementary, Supplementary Material online). This dated species tree provides an evolutionary timescale to evaluate the genomic landscape of phylogenetic discordance across ∼12 my of murine evolution.

### The Genomic Landscape of Phylogenetic Discordance

Limiting the number and nature of the loci used to resolve species relationships is often useful to get an initial picture of the history of speciation across many taxa. However, such targeted approaches may fail to capture the degree of discordance driven by ILS and introgression (Alexander et al. 2017; Chan et al. 2020; Vanderpool et al. 2020; Alda et al. 2021). Our results highlight the limitations of reduced marker-based approaches and the general relationships between phylogenetic patterns and functional attributes of the genome in several interesting ways. Using the house mouse genome annotation, we found that the species tree inferred from only genes or UCEs did not match evolutionary relationships inferred for over 85% of the genome. Although similar frequencies were observed among these three most common trees (Fig. 2), the topology robustly inferred from genes or UCEs was not that common overall and only the third most frequent topology among 10 kb windows genome-wide. This result was driven mainly by discordance among three more closely related (Praomyini) species sampled for this study, which had nodes with low concordance in the UCE species tree (Fig. 1, node J). In the window analysis, each alternate topology of this clade occurred at a frequency of ∼14% while the rest of the topology remained consistent with the species tree (Fig. 2), indicating that the alternate topologies are caused by high levels of ILS at these nodes. Increased discordance at unresolved nodes is a common feature of phylogenomic datasets. These patterns illustrate how extensive discordance can arise even in a small sample of species and underscores that a single inferred species tree often may not capture the history of individual regions of the genome.

Given the fundamental role that recombination should play in shaping patterns of genetic variation within genomes, it is reasonable to assume that patterns of ILS should be broadly shaped by local recombination rate. We did not observe a clear relationship between local recombination rates in mice (*M. musculus*) and the degree of local phylogenetic discordance (i.e. phylogenetic similarity over 5 Mb intervals). However, we did find that regions of high recombination rate tended to be more discordant with the inferred species tree, in line with findings in mammals (Pease and Hahn 2013; Foley et al. 2023; Rivas-Gonzalez et al. 2023) and *Drosophila* (Pease and Hahn 2013). Recombination rates evolve fairly rapidly both within (Kong et al. 2002; Cox et al. 2009; Stapley et al. 2017) and between mammalian species (Jensen-Seaman et al. 2004; Ptak et al. 2005; Stevison et al. 2016; Stapley et al. 2017) likely due, in part, to the high turnover of hotspots due to the changing landscape of binding sites for PRDM9 (Baudat et al. 2010; Singhal et al. 2015). Similar to findings in great apes (Hobolth et al. 2007), our results suggest that high-resolution genetic maps from a single species provide some weak predictive value for understanding broader patterns of species tree discordance. However, these limited estimates may not be predictive of finer-scale patterns in a sample spanning over 12 million years of mammalian evolution (but see Foley et al. 2023). Overall, the evolution of recombination landscapes across closely related species remains an important empirical question in evolutionary genetics (Dapper and Payseur 2017), especially as the generation of chromosome-scale genome assemblies continues to greatly outpace estimates of patterns of recombination within those genomes.

One source of evolution in the recombination map may be changes in synteny. Our reference-guided analyses assume collinearity between *Mus* and the other lineages we are comparing (i.e. no karyotype variation), at least at the window-based scale we are comparing variation. Structural variation, including substantial variation in chromosome numbers, is likely to be widespread in rodents (Stanyon et al. 1999; Yalcin et al. 2011; Romanenko et al. 2012; Keane et al. 2014) and has the potential to skew our results when comparing tree similarity between regions of the genome using multiple species. Generating chromosome-scale assemblies may prove limiting for some non-*Mus* and *Rattus* species given that tissue resources for this group are derived from natural history collections that often lack high molecular weight DNA. Nonetheless, whole genome alignments between mouse and rat indicate high degrees of chromosomal synteny and colinearity (supplementary fig. S6, Supplementary Material online), suggesting that many regions will be colinear in our sample.

Natural selection reduces the effective population size (*N_e_*) of genomic regions through genetic hitchhiking of variation linked to the fixation of positively selected mutations (i.e. selective sweeps; Maynard Smith and Haigh 1974; Kaplan et al. 1989) and the purging of deleterious mutations (i.e. background selection; Charlesworth et al. 1993; Hudson and Kaplan 1995). Thus, variation in parameters dependent on *N_e_*—such as standing levels of nucleotide variation and patterns of ILS—should be reduced by linkage to functional elements subject to selection (Johri et al. 2022). Consistent with this, we observed the lowest rates of local discordance (Fig. 5a) and overall gene tree/species tree discordance (Fig. 5b) near UCEs when compared to all other genomic features we studied. These results suggest that a history of recurrent purifying selection on UCEs (Katzman et al. 2007) strongly reduces patterns of discordance through a persistent local reduction in *N_e_*. In contrast, protein-coding genes showed rates of local discordance that were similar to background levels, even when considering genes rapidly evolving due to positive directional selection (Fig. 5a). However, both classes of genes did show less species tree discordance than background consistent with previous results (Scally et al. 2012; Rivas-Gonzalez et al. 2023), but this effect was much weaker than as observed at UCEs (Fig. 5b). Collectively, these data suggest that the frequency and strength of selection plays an important role in structuring patterns of ILS across the genome over deeper evolutionary timescales.

One practical consequence of this is that phylogenetic inferences based on UCE markers would seem less prone to discordance and may provide cleaner estimates of species tree history than randomly chosen or protein-coding regions. Indeed, windows centered on UCEs have a higher degree of similarity to the species tree than other genomic features (i.e. 17% concordance with the species tree, vs. 13% genome-wide or 15% for protein-coding genes). However, it is worth noting that UCEs are also more likely to provide a potentially misleading underestimate of genome-wide levels of discordance. Given this relationship, species tree inferences based on UCEs should likely not, for example, be extended to related population genetic parameters of interest (e.g. ancestral population sizes, estimates population genetic diversity) and could mislead the reconstruction of protein (see below) or trait evolution across phylogenies (Avise and Robinson 2008; Hahn and Nakhleh 2016; Mendes et al. 2018; Hibbins et al. 2023). Finally, despite the relative ease of generating UCE data, such markers are likely unsuitable for genetic inferences within populations given the pervasive effects of linked selection.

### Discordance and Molecular Evolution

We also found that the choice of tree topology significantly affects the results from various common tests for positive selection. Previous studies have used simulations to show that tree misspecification can lead to incorrect placement of substitutions on branches, possibly leading to spurious results for tests of positive directional selection within empirical datasets (Mendes and Hahn 2016). Here, we use empirical data in mice to document the extent that using local gene trees vs. assuming an overall species tree may shape inferences of positive directional selection on protein-coding sequences.

For each of the three selection tests run (i.e. HyPhy's BUSTED and aBSREL and PAML's M1a vs. M2a), some genes showed evidence of positive selection whether the species tree or gene tree was used. In contrast, many other genes had signatures of positive selection restricted only to a single specification strategy. As expected, genes that were sensitive to specification strategy tended to more discordant with the species tree, while the genes that showed evidence of positive selection regardless of the tree used had levels of discordance comparable to all genes (85%, Fig. 6, numbers in parentheses). This suggests that specifying a potentially incorrect tree (e.g. the species tree when the underlying gene trees are discordant) can often affect inferences of positive selection. The magnitude and direction of these biases were dependent on the underlying model. So-called branch-site models that allow substitution rates to vary among both branches and codon sites, such as HyPhy's BUSTED and aBSREL models, resulted in more genes inferred with evidence for positive selection when using the local gene tree. This suggests that using a single species tree for branch-site tests may reduce the power to detect positive selection. On the other hand, models that only allow rates to vary among sites, such as PAML's M1a vs. M2a test, showed an increase in the number of cases where there was positive selection detected with the species trees but not with the local gene tree. As these inferences are based on empirical data, the actual phylogenetic histories are not known and both specification strategies could result in errors. That said, our findings suggest that phylogenetic discordance may bias results towards spurious increases in *d_N_/d_S_* that mimics positive directional selection in some instances, or loss of power to detect selection in other cases and that the magnitude and direction of these biases vary by model type.

These results have wide-ranging implications for phylogenetics and comparative genomic analysis. First, it is imperative that when testing a specific locus for positive selection, discordance among loci must be accounted for. This is most easily achieved by simply using the gene tree (or other locus type) as input to the test for selection (Good et al. 2013; Mendes and Hahn 2016; Roycroft et al. 2021). However, as Mendes and Hahn (2016) pointed out, this may not completely mask the effects of discordance on substitution rates, as sites within a single gene may still have evolved under different histories because of within-gene recombination. Indeed, we found that tree similarity diminished at scales that were less than the average genomic distance between the beginning and end of a coding sequence in mice (∼37 kb in this data set). Nevertheless, we suggest that starting with an inferred gene tree is advisable whenever possible, followed by a secondary analysis of evidence for within-gene variation in phylogenetic history. Our results also imply that studies of molecular evolution may benefit from approaches that reduce genome-wide levels of discordance, such as through post hoc pruning of species that disproportionately contribute to unresolved nodes.

Incorporating discordance into a comparative framework is not trivial and many comparative genomic methods assume a single species tree that test for changes in substitution rates in a phylogeny (Pollard et al. 2010; Hu et al. 2019; Partha et al. 2019). Even methods that allow the use of different trees for different loci (like PAML and HyPhy) are still commonly applied with a single species tree across loci (Carbone et al. 2014; Foote et al. 2015; van der Valk et al. 2021; Treaster et al. 2023). Our suggestions are on the simple assumption that the results from the local gene tree are more likely to be correct, but, as noted above, this may not always be the case given that errors can also occur during gene tree inference. Still, our results confirm that the use of a single tree for all loci for such tests that rely on accurate estimation of substitution rates are likely to lead to both inaccurate inferences of positive selection. We strongly encourage the use of individual gene trees for such analyses.

## Conclusions

Murine rodents as a study system allow us to use the high-quality *M. musculus* genome to examine fine-scale patterns and effects of phylogenetic discordance along chromosomes. Our analysis reveals how discordance varies with genome biology across evolutionary timescales, as well as the limits of inference inherent to extrapolating information from a single model system to a phylogenetic sample. We also demonstrate how phylogenetic discordance can mislead common tests for selection if only a single species tree is used. Overall, our results emphasize that progress in comparative genomics requires a detailed understanding of the heterogeneous biological signals in phylogenomic datasets.

Our results help illuminate the complexities of phylogenomic datasets and the need to accommodate phylogenetic discordance in genome-wide analyses. Genomic data now dominate the study of both population genetics and phylogenetics, and these once disparate fields are increasingly unified. Species tree phylogenies are an emergent pattern of the genome-wide accumulation of stochastic and directional population-level processes that cannot be fully captured or modeled by a single history (Steenwyk et al. 2023). Importantly, phylogenetic discordance is not limited to closely related populations or species and is expected to leave persistent signals over deep evolutionary timescales (Oliver 2013). In turn, the use of tree-based frameworks for studying evolution (at any timescale) must incorporate the population-level processes that shape phylogenetic discordance. There appear to be relatively few tree-based applications where the use of a single evolutionary history is appropriate. Indeed, failure to account for phylogenetic discordance can lead to spurious inferences of molecular evolution (Fig. 6; Mendes et al. (2016)), trait evolution (Avise and Robinson 2008; Hahn and Nakhleh 2016), and even species diversification (Louca and Pennell 2020). Similar to the need for robust baseline models in population genomic inference (Johri et al. 2022), understanding the causes and landscape of phylogenetic discordance constitutes a critical first step in phylogenomic analysis (Mirarab et al. 2021; Steenwyk et al. 2023).

## Materials and Methods

### Sample Collection and Assembly

We collected genomes from 16 murine species and two other rodents from several sources, including NCBI and several recently sequenced in Kumon et al. (2021) (see supplementary table S1, Supplementary Material online for full list of samples and sources). We also report the genome of *O. typus* (FMNH 230007) from Ethiopia in 2015. While DNA extraction and sequencing on the 10× Genomics platform for *O. typus* is the same as described in Kumon et al. (2021), the library quality for this sample was too low for chromosome level assembly. Here, we instead assembled it into scaffolds with the express purpose of obtaining UCEs for phylogenetic analysis. Adapters and low-quality bases were trimmed from the reads using illumiprocessor (Faircloth 2013), which makes use of functions from trimmomatic (Bolger et al. 2014). All cleaned reads were de novo assembled using ABySS 2.3.1 (Jackman et al. 2017) with a Bloom filter (Bloom 1970) de Bruijn graph. The final *O. typus* scaffold assembly was 2.14GB (N50 = 9,211; L50 = 64,014; E-size = 12,790).

In parallel, for six of these species (see Fig. 1; supplementary table S1, Supplementary Material online), we generated reference-based pseudoassemblies with iterative mapping using an updated version pseudo-it v3.1.1 (Sarver et al. 2017) that incorporates insertion–deletion variation to minimize reference bias in our genome-wide phylogenetic analyses and to maintain collinearity between assemblies (https://github.com/goodest-goodlab/pseudo-it). We used the *M. musculus* (mm10; Mouse Genome Sequencing Consortium 2002) genome as the reference for our pseudoassembly approach. Briefly, pseudo-it maps reads from each sample to the reference genome with BWA (Li 2013), calls variants with GATK HaplotypeCaller (Poplin et al. 2018), and filters SNPs and indels and generates a consensus assembly with bcftools (Danecek et al. 2021). The process is repeated, each time using the previous iteration's consensus assembly as the new reference genome to which reads are mapped. In total, we did three iterations of mapping for each sample.

### UCE Retrieval and Alignment

We first set out to reconstruct a phylogeny of sequenced murine rodents to provide both a general resource for future comparative genomic studies within this important group as well as a time-calibrated phylogeny to frame an in-depth analysis of phylogenetic discordance across a subset of murine whole genomes (see below). We combined our seven recently sequenced genomes with nine publicly available murine genomes as well as the genomes of two nonmurine rodents, the great gerbil (*Rhombomys opimus*; Nilsson et al. 2020) and the Siberian hamster (*Phodopus sungorus*; Moore et al. 2022) as outgroups. We extracted UCEs from each species, plus 1000 flanking bases from each side of the element using the protocols for harvesting loci from genomes and the *M. musculus* UCE probe set provided with phyluce v1.7.1 (Faircloth et al. 2012; Faircloth 2016). In total, we recovered 2,632 unique UCE loci, though not all UCE loci were found in all taxa (supplementary table S1, Supplementary Material online).

We brought the extracted UCE sequences for each species into a consistent orientation using MAFFT v7 (Katoh and Standley 2013) and then aligned them using FSA (Bradley et al. 2009) with the default settings. We trimmed UCE alignments with TrimAl (Capella-Gutierrez et al. 2009) with a gap threshold of 0.5 and otherwise default parameters. We performed alignment quality checks using AMAS (Borowiec 2016). We processed all alignments in parallel with GNU Parallel (Tange 2018).

### Species Tree Reconstruction from UCEs

We constructed a species-level rodent phylogeny with two approaches. First, using the alignments of all UCEs found in four or more taxa (2,632), we reconstructed a maximum likelihood (ML) species tree with IQ-TREE v2.2.1 (Minh, Schmidt et al. 2020). Each UCE alignment was concatenated and partitioned (Chernomor et al. 2016) such that optimal substitution models were inferred for individual UCE loci with ModelFinder (Kalyaanamoorthy et al. 2017). We also reconstructed individual gene trees for each UCE alignment. For all IQ-TREE runs (concatenated or individual loci), we assessed branch support with ultrafast bootstrap approximation (UFBoot) (Hoang et al. 2018) and the corrected approximate likelihood ratio test (SH-aLRT) (Guindon et al. 2010). We collapsed branches in each UCE tree exhibiting less than 10% approximated bootstrap support using the nw_ed function from Newick Utilities (Junier and Zdobnov 2010). We used these trees as input to the quartet summary method ASTRAL-III v5.7.8 (Zhang et al. 2018) to infer a species tree. We generated visualizations of phylogenies with R v4.1.1 (R Core Team 2021) using phytools v1.9-16 (Revell 2012) and the ggtree package v3.14 (Yu et al. 2017; Yu 2020) and its imported functions from ape v5.0 (Paradis and Schliep 2019) and treeio v1.16.2 (Wang et al. 2020).

We then used two methods to assess phylogenetic discordance across the reconstructed species tree. First, we calculated sCF and gCF with IQ-TREE 2 (Minh, Hahn et al. 2020; Minh, Schmidt et al. 2020) to assess levels of phylogenetic discordance between the inferred UCE trees and the concatenated species tree. gCF is calculated for each branch in the species tree as the proportion of UCE trees in which that branch also appears (Baum 2007). sCF represents the proportion of alignment sites concordant with a given species tree branch in a randomized subset of quartets of taxa (Minh, Hahn et al. 2020). We visualized gCF and sCF for each branch in each species tree using methods in R v4.3.0 (Lanfear 2018; R Core Team 2021). Next, we used PhyParts (Smith, Moore et al. 2015) to identify topological conflict between the UCE trees and the species tree from ASTRAL-III. For this analysis, we rooted all trees with *Phodopus sungorus* as the outgroup using the nw_reroot function in the Newick Utilities (Junier and Zdobnov 2010) package and excluded 204 UCE trees that did not contain the outgroup.

### Divergence Time Estimation

We used IQ-TREE 2's (Minh, Schmidt et al. 2020) implementation of least square dating to estimate branch lengths of our species trees in units of absolute time (To et al. 2016). To improve divergence time estimation, we used SortaDate (Smith et al. 2018) to identify a set of 100 UCE loci that exhibit highly clocklike behavior and minimized topological conflict with the concatenated species tree. We applied four node age calibrations (supplementary table S3, Supplementary Material online) as described in Kimura et al. (2015) and Aghova et al. (2018). The origin of core Murinae (node E) was constrained to between 11.1 and 12.3 Ma, following Kimura et al. (2015). Maximum ages were set for Otomyini + Arvicanthini (9.2 Ma, Kimura et al. 2015), *Apodemus* (9.6 Ma, Daxner-Höck 2002), and *Mus* (8.0 Ma, Kimura et al. 2013, 2015). Branch lengths were resampled 100 times to produce CI.

### Genome Window-Based Phylogenetic Analysis

For the second part of our work, we wanted to quantitatively infer phylogenetic discordance across a subset of the murine genomes used to infer the species tree and relate that discordance to other features of the genome, such as recombination rate, proximity to genes, and rates of molecular evolution. To assess the distribution of phylogenetic discordance across rodent genomes, we limited subsequent analyses to *M. musculus* and the pseudoassemblies (see above) of six of the genomes (*M. natalensis*, *H. alleni*, *P. delectorum*, *R. dilectus*, *G. dolichurus*, and *R. soricoides*). *Otomys typus* was excluded from these analyses due to the inadequacy of the library outlined above.

We partitioned these genomes into 10 kilobase (kb) windows based on the coordinates in the reference *M. musculus* genome (mm10; Mouse Genome Sequencing et al. 2002) using bedtools makewindows (Quinlan and Hall 2010). These coordinates were converted between the reference and the consensus sequence for each genome using liftOver (Hinrichs et al. 2006). Note that this method assumes both collinearity of all genomes and similar karyotypes (see Discussion). We then removed windows from the subsequent analyses if (i) 50% or more of the window overlapped with repeat regions from the *M. musculus* reference RepeatMasker (Smit et al. 2013–2015) file downloaded from the UCSC Genome Browser's table browser (Hinrichs et al. 2006) or (ii) 50% or more of the window contained missing data in three or more samples. Overlaps with repeat regions were determined with bedtools coverage (Quinlan and Hall 2010). We then aligned the 10 kb windows with MAFFT (Katoh and Standley 2013), trimmed alignments with trimAl (Capella-Gutierrez et al. 2009), and inferred phylogenies for each with IQ-TREE 2 (Minh, Schmidt et al. 2020) which uses ModelFinder to determine the best substitution model for each window (Kalyaanamoorthy et al. 2017).

To assess patterns of tree similarity between windows on the same chromosome, we used the wRF (Robinson and Foulds 1981; Böcker et al. 2013) distance measure implemented in the phangorn library (Schliep 2011) in R (R Core Team 2021), which compares two trees by finding clades or splits present in one tree but not the other weighted by the missing branch length in each tree for each mismatch and differences in branch length between the cooccurring branches in both trees (Robinson and Foulds 1981). Consequently, the resulting measure of wRF is in units of branch length (i.e. expected number of substitutions per site for ML trees). We compared wRF between trees from windows on the same chromosome to characterize (i) heterogeneity in patterns discordance along the chromosome and (ii) whether tree similarity is correlated with distance between windows. For the second question, we sampled every window on a chromosome at increasing distance (in 10 kb windows) until the distribution of wRF scores for all pairs of windows at that distance was not significantly different (Wilcox test, *P* > 0.01) than that of a sample of 12,000 measures of wRF between randomly selected trees on different chromosomes. We selected 12,000 as the random sample size because it roughly matched the number of windows on the largest chromosome (chromosome 1, *n* = 12,113). We used Snakemake 7 (Mölder et al. 2021) to compute window alignments and trees in parallel.

### Whole Genome Alignment Between Mouse and Rat

To assess how unaccounted for large-scale structural variation may impact our conclusions, we compared the reference mouse and rat genomes. We used minimap2 (Li 2018) to align the mouse (mm10) and rat (rnor6) (Gibbs et al. 2004) genomes to assess the impact of structural variation that spans the divergence of our subset of species used in the discordance analyses. We downloaded the rat reference genome (rnor6) from the UCSC genome browser and for both genomes removed the Y chromosome and all smaller unplaced scaffolds. We then used minimap2 in whole genome alignment mode (-x asm20) to generate a pairwise alignment file from which we calculated alignment segment sizes and the distances between alignment segments. We visualized the alignment as a dot plot using the pafr package in R (https://github.com/dwinter/pafr).

### Recombination Rate and Functional Annotation

We retrieved 10,205 genetic markers generated from a large heterogenous stock of outbred mice (Shifman et al. 2006; Cox et al. 2009) to assess whether phylogenetic discordance along chromosomes was correlated with mouse recombination rates. We converted the physical coordinates of these markers from build 37 (mm9) to build 38 (mm10) of the *M. musculus* genome using liftOver (Hinrichs et al. 2006). We then partitioned the markers into 5Mb windows and estimated local recombination rates defined as the slope of the correlation between the location on the *M. musculus* genetic and physical maps for all markers in the window (White et al. 2009; Kartje et al. 2020). Within each 5Mb window, we calculated wRF distances between the first 10 kb window and every other 10 kb window.

We also compared the chromosome-wide wRF distances to those based on phylogenies from regions around several types of adjacent to genomic features. We retrieved coordinates from 25,753 protein-coding genes annotated in *M. musculus* from Ensembl (release 99; Cunningham et al. 2022), all 3,129 UCEs from the *M. musculus* UCE probe set provided with PHYLUCE (Faircloth et al. 2012; Faircloth 2016), and 9,865 recombination hotspots from Smagulova et al. (2011). The recombination hotspot coordinates were converted between build 37 and build 38 using the liftOver tool (Hinrichs et al. 2006). For each feature, the starting window was the 10 kb window containing the feature's midpoint coordinate. We then calculated wRF between this window and all windows within 5Mb in either direction and for each chromosome compared the slope and wRF distance of windows adjacent to the feature with the same metrics for the whole chromosome. We compared distributions of these measures for each genomic feature with an ANOVA (aov(feature.measure ∼ feature.label)) followed by Tukey's range test (TukeyHSD(anova.result)) to assess differences in means, as implemented in R v4.1.1 (R Core Team 2021).

### Molecular Evolution

To test how tree misspecification affects common model-based analyses of molecular evolution, we retrieved 22,261 coding sequences from *M. musculus* using the longest coding transcript of each gene. Coding coordinates from the *M. musculus* coding sequences were transposed to the new assemblies via liftOver (Hinrichs et al. 2006) and sequences retrieved with bedtools getfasta (Quinlan and Hall 2010). We recovered 17,216 genes that were present in all seven species. Using MACSE (Ranwez et al. 2018), we trimmed nonhomologous regions from each ortholog using trimNonHomologousFragments, aligned the orthologs using alignSequences, and trimmed the aligned sequences with trimAlignment to remove unaligned flanking regions. Finally, we manually filtered the alignments using the following (nonmutually exclusive) criteria: 3,368 alignments were removed during filtering for gapped sites, 3,132 alignments had a premature stop codon in at least one species, 1,571 alignments had only three or fewer unique sequences among the seven species, and 78 alignments were shorter than 100 bp. After filtering, 12,559 total alignments for tree reconstruction and inference of selection.

We then used IQ-TREE 2 (Minh, Schmidt et al. 2020) to reconstruct a single species tree from concatenation of all gene alignments, as well as gene trees for each individual alignment. This species tree from coding regions matches the topologies of these species inferred by concatenation of UCEs in the previous section. Next, we ran several tests that use both coding alignments and a tree to infer positive selection: PAML's M1a vs. M2a test (Yang 2007), HyPhy's aBSREL model (Smith, Wertheim et al. 2015), and HyPhy's BUSTED model (Murrell et al. 2015). We ran each test twice on each gene, once using the species tree derived from concatenated data, and once using the tree estimated for that gene. For the HyPhy models, no target branch was selected, meaning all branches in the input phylogeny were tested.

The end point of each of these three tests is a *P*-value, which lets us assess whether a model that allows for positively selected sites fits better than a model that does not. For M1a vs. M2a, we obtained the *P*-value manually by first performing a likelihood ratio test to determine genes under selection by calculating 2*(lnlM1a–lnlM2a). The *P*-value of this likelihood ratio is then retrieved from a one-tailed chi-square distribution with two degrees of freedom (Yang 2007). For BUSTED and aBSREL, *P*-values are computed automatically during the test using similar likelihood ratios. For the M1a vs. M2a and BUSTED tests, a single *P*-value is computed for each gene. *P*-values were adjusted by correcting for false discovery rates (Benjamini and Hochberg 1995; Yekutieli and Benjamini 1999) using the “fdr” method in the p.adjust() function in R (R Core Team 2021), and we categorized a gene as being positively selected if its adjusted *P*-value was <0.01. For the aBSREL test, a *P*-value is generated for each branch in the input gene tree. aBSREL corrects for multiple testing internally across branches using the Holm–Bonferroni procedure (Holm 1979; Pond et al. 2005). We further correct the *P*-values across genes with the Bonferroni method and classify a gene as having experienced positive selection if one or more branches has a *P*-value <0.01 after all corrections. We used Snakemake 7 (Mölder et al. 2021) to compute coding alignments, trees, and selection tests in parallel.

## Supplementary Material

evaf017_Supplementary_Data

## Data Availability

For the six previously assembled genomes (see supplementary table S1, Supplementary Material online), all raw reads and assemblies are available as an NCBI BioProject (Accession Number PRJNA669840). The reads and assembly for *Otomys* typus, pseudoassemblies for the six other new samples, and locus alignments (UCEs, genes, and genomic windows) are available on Dryad (https://doi.org/10.5061/dryad.866t1g1wq.). All code and summary data for this project are deposited on github (https://github.com/gwct/murine-discordance).

## References

[evaf017-B1] Aghova T, Kimura Y, Bryja J, Dobigny G, Granjon L, Kergoat GJ. Fossils know it best: using a new set of fossil calibrations to improve the temporal phylogenetic framework of murid rodents (Rodentia: Muridae). Mol Phylogenet Evol. 2018:128:98–111. 10.1016/j.ympev.2018.07.017. 30030180

[evaf017-B2] Alda F, Ludt WB, Elias DJ, McMahan CD, Chakrabarty P. Comparing ultraconserved elements and exons for phylogenomic analyses of middle American cichlids: when data agree to disagree. Genome Biol Evol. 2021:13(8):evab161. 10.1093/gbe/evab161. 34272856 PMC8369075

[evaf017-B3] Alexander AM, Su YC, Oliveros CH, Olson KV, Travers SL, Brown RM. Genomic data reveals potential for hybridization, introgression, and incomplete lineage sorting to confound phylogenetic relationships in an adaptive radiation of narrow-mouth frogs. Evolution. 2017:71(2):475–488. 10.1111/evo.13133. 27886369

[evaf017-B4] Avise JC, Robinson TJ. Hemiplasy: a new term in the lexicon of phylogenetics. Syst Biol. 2008:57(3):503–507. 10.1080/10635150802164587. 18570042

[evaf017-B5] Baudat F, Buard J, Grey C, Fledel-Alon A, Ober C, Przeworski M, Coop G, de Massy B. PRDM9 is a major determinant of meiotic recombination hotspots in humans and mice. Science. 2010:327(5967):836–840. 10.1126/science.1183439. 20044539 PMC4295902

[evaf017-B6] Baum DA . Concordance trees, concordance factors, and the exploration of reticulate genealogy. TAXON. 2007:56(2):417–426. 10.1002/tax.562013.

[evaf017-B7] Benjamini Y, Hochberg Y. Controlling the false discovery rate: a practical and powerful approach to multiple testing. J R Stat Soc: Ser B (Methodol). 1995:57(1):289–300. 10.1111/j.2517-6161.1995.tb02031.x.

[evaf017-B8] Bloom BH . Space/time trade-offs in hash coding with allowable errors. Commun ACM. 1970:13(7):422–426. 10.1145/362686.362692.

[evaf017-B9] Böcker S, Canzar S, Gunnar WK. The generalized robinson-foulds metric. In: Darling A, Stoye J, editors. Algorithms in bioinformatics. Berlin, Heidelberg: Springer; 2013. p. 156–169.

[evaf017-B10] Bolger AM, Lohse M, Usadel B. Trimmomatic: a flexible trimmer for Illumina sequence data. Bioinformatics. 2014:30(15):2114–2120. 10.1093/bioinformatics/btu170. 24695404 PMC4103590

[evaf017-B11] Borowiec ML . AMAS: a fast tool for alignment manipulation and computing of summary statistics. PeerJ. 2016:4:e1660. 10.7717/peerj.1660. 26835189 PMC4734057

[evaf017-B12] Bradley RK, Roberts A, Smoot M, Juvekar S, Do J, Dewey C, Holmes I, Pachter L. Fast statistical alignment. PLoS Comput Biol. 2009:5(5):e1000392. 10.1371/journal.pcbi.1000392. 19478997 PMC2684580

[evaf017-B13] Capella-Gutierrez S, Silla-Martinez JM, Gabaldon T. Trimal: a tool for automated alignment trimming in large-scale phylogenetic analyses. Bioinformatics. 2009:25(15):1972–1973. 10.1093/bioinformatics/btp348. 19505945 PMC2712344

[evaf017-B14] Carbone L, Harris RA, Gnerre S, Veeramah KR, Lorente-Galdos B, Huddleston J, Meyer TJ, Herrero J, Roos C, Aken B, et al Gibbon genome and the fast karyotype evolution of small apes. Nature. 2014:513(7517):195–201. 10.1038/nature13679. 25209798 PMC4249732

[evaf017-B15] Chan KO, Hutter CR, Wood PL Jr, Grismer LL, Brown RM. Target-capture phylogenomics provide insights on gene and species tree discordances in old world treefrogs (Anura: Rhacophoridae). Proc Biol Sci. 2020:287(1940):20202102. 10.1098/rspb.2020.2102. 33290680 PMC7739936

[evaf017-B16] Charlesworth B, Morgan MT, Charlesworth D. The effect of deleterious mutations on neutral molecular variation. Genetics. 1993:134(4):1289–1303. 10.1093/genetics/134.4.1289. 8375663 PMC1205596

[evaf017-B17] Chernomor O, von Haeseler A, Minh BQ. Terrace aware data structure for phylogenomic inference from supermatrices. Syst Biol. 2016:65(6):997–1008. 10.1093/sysbio/syw037. 27121966 PMC5066062

[evaf017-B18] Christmas MJ, Kaplow IM, Genereux DP, Dong MX, Hughes GM, Li X, Sullivan PF, Hindle AG, Andrews G, Armstrong JC, et al Evolutionary constraint and innovation across hundreds of placental mammals. Science. 2023:380(6643):eabn3943. 10.1126/science.abn3943. 37104599 PMC10250106

[evaf017-B19] Cox A, Ackert-Bicknell CL, Dumont BL, Ding Y, Bell JT, Brockmann GA, Wergedal JE, Bult C, Paigen B, Flint J, et al A new standard genetic map for the laboratory mouse. Genetics. 2009:182(4):1335–1344. 10.1534/genetics.109.105486. 19535546 PMC2728870

[evaf017-B20] Cunningham F, Allen JE, Allen J, Alvarez-Jarreta J, Amode MR, Armean IM, Austine-Orimoloye O, Azov AG, Barnes I, Bennett R, et al Ensembl 2022. Nucleic Acids Res. 2022:50(D1):D988–D995. 10.1093/nar/gkab1049. 34791404 PMC8728283

[evaf017-B21] Danecek P, Bonfield JK, Liddle J, Marshall J, Ohan V, Pollard MO, Whitwham A, Keane T, McCarthy SA, Davies RM, et al Twelve years of SAMtools and BCFtools. Gigascience. 2021:10(2):giab008. 10.1093/gigascience/giab008. 33590861 PMC7931819

[evaf017-B22] Dapper AL, Payseur BA. Connecting theory and data to understand recombination rate evolution. Philos Trans R Soc Lond B Biol Sci. 2017:372(1736):20160469. 10.1098/rstb.2016.0469. 29109228 PMC5698627

[evaf017-B23] Daxner-Höck G . Cricetodon meini and other rodents from Mühlbach and Grund, Lower Austria (Middle Miocene, late MN5). Annalen des Naturhistorischen Museums in Wien. Serie A für Mineralogie und Petrographie, Geologie und Paläontologie, Anthropologie und Prähistorie. 2002:104:267–291. https://www.jstor.org/stable/41702046.

[evaf017-B24] Degnan JH, Rosenberg NA. Discordance of species trees with their most likely gene trees. PLoS Genet. 2006:2(5):e68. 10.1371/journal.pgen.0020068. 16733550 PMC1464820

[evaf017-B25] Edwards SV . Is a new and general theory of molecular systematics emerging? Evolution. 2009:63(1):1–19. 10.1111/j.1558-5646.2008.00549.x. 19146594

[evaf017-B26] Faircloth BC . 2013. Ilumiprocessor: a trimmomatic wrapper for paralleladapter and quality trimming. 2.0.3. 10.6079/J9ILL.

[evaf017-B27] Faircloth BC . PHYLUCE is a software package for the analysis of conserved genomic loci. Bioinformatics. 2016:32(5):786–788. 10.1093/bioinformatics/btv646. 26530724

[evaf017-B28] Faircloth BC, McCormack JE, Crawford NG, Harvey MG, Brumfield RT, Glenn TC. Ultraconserved elements anchor thousands of genetic markers spanning multiple evolutionary timescales. Syst Biol. 2012:61(5):717–726. 10.1093/sysbio/sys004. 22232343

[evaf017-B29] Feng S, Bai M, Rivas-Gonzalez I, Li C, Liu S, Tong Y, Yang H, Chen G, Xie D, Sears KE, et al Incomplete lineage sorting and phenotypic evolution in marsupials. Cell. 2022:185(10):1646–1660.e1618. 10.1016/j.cell.2022.03.034. 35447073 PMC9200472

[evaf017-B30] Ferreira MS, Jones MR, Callahan CM, Farelo L, Tolesa Z, Suchentrunk F, Boursot P, Mills LS, Alves PC, Good JM, et al The legacy of recurrent introgression during the radiation of hares. Syst Biol. 2021:70(3):593–607. 10.1093/sysbio/syaa088. 33263746 PMC8048390

[evaf017-B31] Foley NM, Harris AJ, Bredemeyer KR, Ruedi M, Puechmaille SJ, Teeling EC, Criscitiello MF, Murphy WJ. Karyotypic stasis and swarming influenced the evolution of viral tolerance in a species-rich bat radiation. Cell Genom. 2024:4(2):100482. 10.1016/j.xgen.2023.100482. 38237599 PMC10879000

[evaf017-B32] Foley NM, Mason VC, Harris AJ, Bredemeyer KR, Damas J, Lewin HA, Eizirik E, Gatesy J, Karlsson EK, Lindblad-Toh K, et al A genomic timescale for placental mammal evolution. Science. 2023:380(6643):eabl8189. 10.1126/science.abl8189. 37104581 PMC10233747

[evaf017-B33] Fontaine MC, Pease JB, Steele A, Waterhouse RM, Neafsey DE, Sharakhov IV, Jiang X, Hall AB, Catteruccia F, Kakani E, et al Mosquito genomics. Extensive introgression in a malaria vector species complex revealed by phylogenomics. Science. 2015:347(6217):1258524. 10.1126/science.1258524. 25431491 PMC4380269

[evaf017-B34] Foote AD, Liu Y, Thomas GW, Vinar T, Alfoldi J, Deng J, Dugan S, van Elk CE, Hunter ME, Joshi V, et al Convergent evolution of the genomes of marine mammals. Nat Genet. 2015:47(3):272–275. 10.1038/ng.3198. 25621460 PMC4644735

[evaf017-B35] Gable SM, Byars MI, Literman R, Tollis M. A genomic perspective on the evolutionary diversification of turtles. Syst Biol. 2022:71(6):1331–1347. 10.1093/sysbio/syac019. 35253878

[evaf017-B36] Gibbs RA, Weinstock GM, Metzker ML, Muzny DM, Sodergren EJ, Scherer S, Scott G, Steffen D, Worley KC, Burch PE, et al Genome sequence of the Brown Norway rat yields insights into mammalian evolution. Nature. 2004:428(6982):493–521. 10.1038/nature02426. 15057822

[evaf017-B37] Good JM, Wiebe V, Albert FW, Burbano HA, Kircher M, Green RE, Halbwax M, Andre C, Atencia R, Fischer A, et al Comparative population genomics of the ejaculate in humans and the great apes. Mol Biol Evol. 2013:30(4):964–976. 10.1093/molbev/mst005. 23329688

[evaf017-B38] Green RE, Krause J, Briggs AW, Maricic T, Stenzel U, Kircher M, Patterson N, Li H, Zhai W, Fritz MH, et al A draft sequence of the Neandertal genome. Science. 2010:328(5979):710–722. 10.1126/science.1188021. 20448178 PMC5100745

[evaf017-B39] Guindon S, Dufayard JF, Lefort V, Anisimova M, Hordijk W, Gascuel O. New algorithms and methods to estimate maximum-likelihood phylogenies: assessing the performance of PhyML 3.0. Syst Biol. 2010:59(3):307–321. 10.1093/sysbio/syq010. 20525638

[evaf017-B40] Hahn MW, Nakhleh L. Irrational exuberance for resolved species trees. Evolution. 2016:70(1):7–17. 10.1111/evo.12832. 26639662

[evaf017-B41] He B, Zhao Y, Su C, Lin G, Wang Y, Li L, Ma J, Yang Q, Hao J. Phylogenomics reveal extensive phylogenetic discordance due to incomplete lineage sorting following the rapid radiation of alpine butterflies (Papilionidae: Parnassius). Syst Entomol. 2023:48(4): 585–599. 10.1111/syen.12592.

[evaf017-B42] Hibbins MS, Breithaupt LC, Hahn MW. Phylogenomic comparative methods: accurate evolutionary inferences in the presence of gene tree discordance. Proc Natl Acad Sci U S A. 2023:120(22):e2220389120. 10.1073/pnas.2220389120. 37216509 PMC10235958

[evaf017-B43] Hinrichs AS, Karolchik D, Baertsch R, Barber GP, Bejerano G, Clawson H, Diekhans M, Furey TS, Harte RA, Hsu F, et al The UCSC genome browser database: update 2006. Nucleic Acids Res. 2006:34(90001):D590–D598. 10.1093/nar/gkj144. 16381938 PMC1347506

[evaf017-B44] Hoang DT, Chernomor O, von Haeseler A, Minh BQ, Vinh LS. UFBoot2: improving the ultrafast bootstrap approximation. Mol Biol Evol. 2018:35(2):518–522. 10.1093/molbev/msx281. 29077904 PMC5850222

[evaf017-B45] Hobolth A, Christensen OF, Mailund T, Schierup MH. Genomic relationships and speciation times of human, chimpanzee, and gorilla inferred from a coalescent hidden Markov model. PLoS Genet. 2007:3(2):e7. 10.1371/journal.pgen.0030007. 17319744 PMC1802818

[evaf017-B46] Holm S . A simple sequentially rejective multiple test procedure. Scandinavian J Stat. 1979:6(2):65–70. https://www.jstor.org/stable/4615733.

[evaf017-B47] Hu Z, Sackton TB, Edwards SV, Liu JS. Bayesian detection of convergent rate changes of conserved noncoding elements on phylogenetic trees. Mol Biol Evol. 2019:36(5):1086–1100. 10.1093/molbev/msz049. 30851112 PMC6501877

[evaf017-B48] Hudson RR . Testing the constant-rate neutral allele model with protein sequence data. Evolution. 1983:37(1):203–217. 10.2307/2408186. 28568026

[evaf017-B49] Hudson RR, Kaplan NL. The coalescent process in models with selection and recombination. Genetics. 1988:120(3):831–840. 10.1093/genetics/120.3.831. 3147214 PMC1203560

[evaf017-B50] Hudson RR, Kaplan NL. Deleterious background selection with recombination. Genetics. 1995:141(4):1605–1617. 10.1093/genetics/141.4.1605. 8601498 PMC1206891

[evaf017-B51] Huson DH, Klöpper T, Lockhart PJ, Steel MA. Reconstruction of reticulate networks from gene trees. In: Miyano S, Mesirov J, Kasif S, Istrail S, Pevzner PA, Waterman M, editors. Research in computational molecular biology. RECOMB 2005. Lecture notes in computer science. Berlin, Heidelberg: Springer; 2005. p. 233–249.

[evaf017-B52] Jackman SD, Vandervalk BP, Mohamadi H, Chu J, Yeo S, Hammond SA, Jahesh G, Khan H, Coombe L, Warren RL, et al ABySS 2.0: resource-efficient assembly of large genomes using a bloom filter. Genome Res. 2017:27(5):768–777. 10.1101/gr.214346.116. 28232478 PMC5411771

[evaf017-B53] Jarvis ED, Mirarab S, Aberer AJ, Li B, Houde P, Li C, Ho SY, Faircloth BC, Nabholz B, Howard JT, et al Whole-genome analyses resolve early branches in the tree of life of modern birds. Science. 2014:346(6215):1320–1331. 10.1126/science.1253451. 25504713 PMC4405904

[evaf017-B54] Jensen-Seaman MI, Furey TS, Payseur BA, Lu Y, Roskin KM, Chen CF, Thomas MA, Haussler D, Jacob HJ. Comparative recombination rates in the rat, mouse, and human genomes. Genome Res. 2004:14(4):528–538. 10.1101/gr.1970304. 15059993 PMC383296

[evaf017-B55] Johri P, Aquadro CF, Beaumont M, Charlesworth B, Excoffier L, Eyre-Walker A, Keightley PD, Lynch M, McVean G, Payseur BA, et al Recommendations for improving statistical inference in population genomics. PLoS Biol. 2022:20(5):e3001669. 10.1371/journal.pbio.3001669. 35639797 PMC9154105

[evaf017-B56] Jones MR, Mills LS, Alves PC, Callahan CM, Alves JM, Lafferty DJR, Jiggins FM, Jensen JD, Melo-Ferreira J, Good JM. Adaptive introgression underlies polymorphic seasonal camouflage in snowshoe hares. Science. 2018:360(6395):1355–1358. 10.1126/science.aar5273. 29930138

[evaf017-B57] Junier T, Zdobnov EM. The Newick utilities: high-throughput phylogenetic tree processing in the UNIX shell. Bioinformatics. 2010:26(13):1669–1670. 10.1093/bioinformatics/btq243. 20472542 PMC2887050

[evaf017-B58] Kalyaanamoorthy S, Minh BQ, Wong TKF, von Haeseler A, Jermiin LS. ModelFinder: fast model selection for accurate phylogenetic estimates. Nat Methods. 2017:14(6):587–589. 10.1038/nmeth.4285. 28481363 PMC5453245

[evaf017-B59] Kaplan NL, Hudson RR, Langley CH. The “hitchhiking effect” revisited. Genetics. 1989:123(4):887–899. 10.1093/genetics/123.4.887. 2612899 PMC1203897

[evaf017-B60] Kartje ME, Jing P, Payseur BA. Weak correlation between nucleotide variation and recombination rate across the house mouse genome. Genome Biol Evol. 2020:12(4):293–299. 10.1093/gbe/evaa045. 32108880 PMC7186785

[evaf017-B61] Katoh K, Standley DM. MAFFT multiple sequence alignment software version 7: improvements in performance and usability. Mol Biol Evol. 2013:30(4):772–780. 10.1093/molbev/mst010. 23329690 PMC3603318

[evaf017-B62] Katzman S, Kern AD, Bejerano G, Fewell G, Fulton L, Wilson RK, Salama SR, Haussler D. Human genome ultraconserved elements are ultraselected. Science. 2007:317(5840):915. 10.1126/science.1142430. 17702936

[evaf017-B63] Keane TM, Wong K, Adams DJ, Flint J, Reymond A, Yalcin B. Identification of structural variation in mouse genomes. Front Genet. 2014:5:192. 10.3389/fgene.2014.00192. 25071822 PMC4079067

[evaf017-B64] Kimura Y, Hawkins MT, McDonough MM, Jacobs LL, Flynn LJ. Corrected placement of Mus-Rattus fossil calibration forces precision in the molecular tree of rodents. Sci Rep. 2015:5(1):14444. 10.1038/srep14444. 26411391 PMC4585935

[evaf017-B65] Kimura Y, Jacobs LL, Cerling TE, Uno KT, Ferguson KM, Flynn LJ, Patnaik R. Fossil mice and rats show isotopic evidence of niche partitioning and change in dental ecomorphology related to dietary shift in Late Miocene of Pakistan. PLoS One. 2013:8(8):e69308. 10.1371/journal.pone.0069308. 23936324 PMC3732283

[evaf017-B66] Kong A, Gudbjartsson DF, Sainz J, Jonsdottir GM, Gudjonsson SA, Richardsson B, Sigurdardottir S, Barnard J, Hallbeck B, Masson G, et al A high-resolution recombination map of the human genome. Nat Genet. 2002:31(3):241–247. 10.1038/ng917. 12053178

[evaf017-B67] Kowalczyk A, Meyer WK, Partha R, Mao W, Clark NL, Chikina M. RERconverge: an R package for associating evolutionary rates with convergent traits. Bioinformatics. 2019:35(22):4815–4817. 10.1093/bioinformatics/btz468. 31192356 PMC6853647

[evaf017-B68] Kulathinal RJ, Stevison LS, Noor MA. The genomics of speciation in Drosophila: diversity, divergence, and introgression estimated using low-coverage genome sequencing. PLoS Genet. 2009:5(7):e1000550. 10.1371/journal.pgen.1000550. 19578407 PMC2696600

[evaf017-B69] Kumon T, Ma J, Akins RB, Stefanik D, Nordgren CE, Kim J, Levine MT, Lampson MA. Parallel pathways for recruiting effector proteins determine centromere drive and suppression. Cell. 2021:184(19):4904–4918.e4911. 10.1016/j.cell.2021.07.037. 34433012 PMC8448984

[evaf017-B70] Lanfear R . 2018. Calculating and interpreting gene- and site-concordance factors in phylogenomics. The Lanfear Lab @ ANU2018. [accessed Sept 20, 2021]. http://www.robertlanfear.com/blog/files/concordance_factors.html.

[evaf017-B71] Lecompte E, Aplin K, Denys C, Catzeflis F, Chades M, Chevret P. Phylogeny and biogeography of African Murinae based on mitochondrial and nuclear gene sequences, with a new tribal classification of the subfamily. BMC Evol Biol. 2008:8(1):199. 10.1186/1471-2148-8-199. 18616808 PMC2490707

[evaf017-B72] Lewontin RC, Birch LC. Hybridization as a source of variation for adaptation to new environments. Evolution. 1966:20(3):315–336. 10.2307/2406633. 28562982

[evaf017-B73] Li H . Aligning sequence reads, clone sequences and assembly contigs with BWA-MEM. bioRxiv 1303.3997. 10.48550/arXiv.1303.3997, 16 March 2013, preprint: not peer reviewed .

[evaf017-B74] Li H . Minimap2: pairwise alignment for nucleotide sequences. Bioinformatics. 2018:34(18):3094–3100. 10.1093/bioinformatics/bty191. 29750242 PMC6137996

[evaf017-B75] Liu X, Wei F, Li M, Jiang X, Feng Z, Hu J. Molecular phylogeny and taxonomy of wood mice (genus Apodemus Kaup, 1829) based on complete mtDNA cytochrome b sequences, with emphasis on Chinese species. Mol Phylogenet Evol. 2004:33(1):1–15. 10.1016/j.ympev.2004.05.011. 15324834

[evaf017-B76] Lopes F, Oliveira LR, Kessler A, Beux Y, Crespo E, Cardenas-Alayza S, Majluf P, Sepulveda M, Brownell RL, Franco-Trecu V, et al Phylogenomic discordance in the eared seals is best explained by incomplete lineage sorting following explosive radiation in the southern hemisphere. Syst Biol. 2021:70(4):786–802. 10.1093/sysbio/syaa099. 33367817

[evaf017-B77] Louca S, Pennell MW. Extant timetrees are consistent with a myriad of diversification histories. Nature. 2020:580(7804):502–505. 10.1038/s41586-020-2176-1. 32322065

[evaf017-B78] Lundrigan BL, Jansa SA, Tucker PK. Phylogenetic relationships in the genus Mus, based on paternally, maternally, and biparentally inherited characters. Syst Biol. 2002:51(3):410–431. 10.1080/10635150290069878. 12079642

[evaf017-B79] Maddison WP . Gene trees in species trees. Syst Biol. 1997:46(3):523–536. 10.1093/sysbio/46.3.523.

[evaf017-B80] Martin Y, Gerlach G, Schlotterer C, Meyer A. Molecular phylogeny of European muroid rodents based on complete cytochrome b sequences. Mol Phylogenet Evol. 2000:16(1):37–47. 10.1006/mpev.1999.0760. 10877938

[evaf017-B81] Maynard Smith J, Haigh J. The hitch-hiking effect of a favourable gene. Genet Res. 1974:23(1):23–35. 10.1017/S0016672300014634. 4407212

[evaf017-B82] McKenzie PF, Eaton DAR. The multispecies coalescent in space and time. bioRxiv 233395. 10.1101/2020.08.02.233395, 03 August 2020, preprint: not peer reviewed.

[evaf017-B83] Mendes FK, Fuentes-Gonzalez JA, Schraiber JG, Hahn MW. A multispecies coalescent model for quantitative traits. Elife. 2018:7:e36482. 10.7554/eLife.36482. 29969096 PMC6092125

[evaf017-B84] Mendes FK, Hahn MW. Gene tree discordance causes apparent substitution rate variation. Syst Biol. 2016:65(4):711–721. 10.1093/sysbio/syw018. 26927960

[evaf017-B85] Mendes FK, Hahn Y, Hahn MW. Gene tree discordance can generate patterns of diminishing convergence over time. Mol Biol Evol. 2016:33(12):3299–3307. 10.1093/molbev/msw197. 27634870

[evaf017-B86] Mendes FK, Livera AP, Hahn MW. The perils of intralocus recombination for inferences of molecular convergence. Philos Trans R Soc Lond B Biol Sci. 2019:374(1777):20180244. 10.1098/rstb.2018.0244. 31154973 PMC6560264

[evaf017-B87] Mikula O, Nicolas V, Šumbera R, Konečný A, Denys C, Verheyen E, Bryjová A, Lemmon AR, Lemmon EM, Bryja J. Nuclear phylogenomics, but not mitogenomics, resolves the most successful Late Miocene radiation of African mammals (Rodentia: Muridae: Arvicanthini). Mol Phylogenet Evol. 2021:157:107069. 10.1016/j.ympev.2021.107069. 33421615

[evaf017-B88] Minh BQ, Hahn MW, Lanfear R. New methods to calculate concordance factors for phylogenomic datasets. Mol Biol Evol. 2020:37(9):2727–2733. 10.1093/molbev/msaa106. 32365179 PMC7475031

[evaf017-B89] Minh BQ, Schmidt HA, Chernomor O, Schrempf D, Woodhams MD, von Haeseler A, Lanfear R. IQ-TREE 2: new models and efficient methods for phylogenetic inference in the genomic era. Mol Biol Evol. 2020:37(5):1530–1534. 10.1093/molbev/msaa015. 32011700 PMC7182206

[evaf017-B90] Mirarab S, Nakhleh L, Warnow T. Multispecies coalescent: theory and applications in phylogenetics. Annu Rev Ecol Evol Syst. 2021:52(1):247–268. 10.1146/annurev-ecolsys-012121-095340.

[evaf017-B91] Mölder F, Jablonski KP, Letcher B, Hall MB, Tomkins-Tinch CH, Sochat V, Forster J, Lee S, Twardziok SO, Kanitz A, et al Sustainable data analysis with Snakemake. F1000Research 2021:10:33. 10.12688/f1000research.29032.2. 34035898 PMC8114187

[evaf017-B92] Moore EC, Thomas GWC, Mortimer S, Kopania EEK, Hunnicutt KE, Clare-Salzler ZJ, Larson EL, Good JM. The evolution of widespread recombination suppression on the dwarf hamster (phodopus) X chromosome. Genome Biol Evol. 2022:14(6):evac080. 10.1093/gbe/evac080. 35642315 PMC9185382

[evaf017-B93] Murrell B, Weaver S, Smith MD, Wertheim JO, Murrell S, Aylward A, Eren K, Pollner T, Martin DP, Smith DM, et al Gene-wide identification of episodic selection. Mol Biol Evol. 2015:32(5):1365–1371. 10.1093/molbev/msv035. 25701167 PMC4408417

[evaf017-B94] Nilsson P, Solbakken MH, Schmid BV, Orr RJS, Lv R, Cui Y, Song Y, Zhang Y, Baalsrud HT, Torresen OK, et al The genome of the great gerbil reveals species-specific duplication of an MHCII gene. Genome Biol Evol. 2020:12(2):3832–3849. 10.1093/gbe/evaa008. 31971556 PMC7046166

[evaf017-B95] Oliver JC . Microevolutionary processes generate phylogenomic discordance at ancient divergences. Evolution. 2013:67(6):1823–1830. 10.1111/evo.12047. 23730773

[evaf017-B96] Pagès M, Fabre P-H, Chaval Y, Mortelliti A, Nicolas V, Wells K, Michaux JR, Lazzari V. Molecular phylogeny of South-East Asian arboreal murine rodents. Zoologica Scripta. 2016:45(4):349–364. 10.1111/zsc.12161.

[evaf017-B97] Pamilo P, Nei M. Relationships between gene trees and species trees. Mol Biol Evol. 1988:5(5):568–583. 10.1093/oxfordjournals.molbev.a040517. 3193878

[evaf017-B98] Paradis E, Schliep K. Ape 5.0: an environment for modern phylogenetics and evolutionary analyses in R. Bioinformatics. 2019:35(3):526–528. 10.1093/bioinformatics/bty633. 30016406

[evaf017-B99] Partha R, Kowalczyk A, Clark NL, Chikina M. Robust method for detecting convergent shifts in evolutionary rates. Mol Biol Evol. 2019:36(8):1817–1830. 10.1093/molbev/msz107. 31077321 PMC6657723

[evaf017-B100] Pease JB, Haak DC, Hahn MW, Moyle LC. Phylogenomics reveals three sources of adaptive variation during a rapid radiation. PLoS Biol. 2016:14(2):e1002379. 10.1371/journal.pbio.1002379. 26871574 PMC4752443

[evaf017-B101] Pease JB, Hahn MW. More accurate phylogenies inferred from low-recombination regions in the presence of incomplete lineage sorting. Evolution. 2013:67(8):2376–2384. 10.1111/evo.12118. 23888858 PMC3929462

[evaf017-B102] Platt RN 2nd, Vandewege MW, Ray DA. Mammalian transposable elements and their impacts on genome evolution. Chromosome Res. 2018:26(1-2):25–43. 10.1007/s10577-017-9570-z. 29392473 PMC5857283

[evaf017-B103] Pollard KS, Hubisz MJ, Rosenbloom KR, Siepel A. Detection of nonneutral substitution rates on mammalian phylogenies. Genome Res. 2010:20(1):110–121. 10.1101/gr.097857.109. 19858363 PMC2798823

[evaf017-B104] Pond SL, Frost SD, Muse SV. Hyphy: hypothesis testing using phylogenies. Bioinformatics. 2005:21(5):676–679. 10.1093/bioinformatics/bti079. 15509596

[evaf017-B105] Poplin R, Ruano-Rubio V, DePristo MA, Fennell TJ, Carneiro MO, Auwera GAVd, Kling DE, Gauthier LD, Levy-Moonshine A, Roazen D, et al Scaling accurate genetic variant discovery to tens of thousands of samples. bioRxiv 201178. 10.1101/201178, 24 July 2018, preprint: not peer reviewed.

[evaf017-B106] Ptak SE, Hinds DA, Koehler K, Nickel B, Patil N, Ballinger DG, Przeworski M, Frazer KA, Paabo S. Fine-scale recombination patterns differ between chimpanzees and humans. Nat Genet. 2005:37(4):429–434. 10.1038/ng1529. 15723063

[evaf017-B107] Quinlan AR, Hall IM. BEDTools: a flexible suite of utilities for comparing genomic features. Bioinformatics. 2010:26(6):841–842. 10.1093/bioinformatics/btq033. 20110278 PMC2832824

[evaf017-B108] Ranwez V, Douzery EJP, Cambon C, Chantret N, Delsuc F. MACSE v2: toolkit for the alignment of coding sequences accounting for frameshifts and stop codons. Mol Biol Evol. 2018:35(10):2582–2584. 10.1093/molbev/msy159. 30165589 PMC6188553

[evaf017-B109] R Core Team . R: a language and environment for statistical computing. Vienna, Austria; 2021.

[evaf017-B110] Revell LJ . Phytools: an R package for phylogenetic comparative biology (and other things). Methods Ecol Evol. 2012:3(2):217–223. 10.1111/j.2041-210X.2011.00169.x.

[evaf017-B111] Rivas-Gonzalez I, Rousselle M, Li F, Zhou L, Dutheil JY, Munch K, Shao Y, Wu D, Schierup MH, Zhang G. Pervasive incomplete lineage sorting illuminates speciation and selection in primates. Science. 2023:380(6648):eabn4409. 10.1126/science.abn4409. 37262154

[evaf017-B112] Robinson DF, Foulds LR. Comparison of phylogenetic trees. Math Biosci. 1981:53(1-2):131–147. 10.1016/0025-5564(81)90043-2.

[evaf017-B113] Romanenko SA, Perelman PL, Trifonov VA, Graphodatsky AS. Chromosomal evolution in Rodentia. Heredity (Edinb). 2012:108(1):4–16. 10.1038/hdy.2011.110. 22086076 PMC3238120

[evaf017-B114] Rosenberg NA . The probability of topological concordance of gene trees and species trees. Theor Popul Biol. 2002:61(2):225–247. 10.1006/tpbi.2001.1568. 11969392

[evaf017-B115] Rowe KC, Achmadi AS, Fabre P-H, Schenk JJ, Steppan SJ, Esselstyn JA. Oceanic islands of wallacea as a source for dispersal and diversification of murine rodents. J Biogeogr. 2019:46(12):2752–2768. 10.1111/jbi.13720.

[evaf017-B116] Roycroft E, Achmadi A, Callahan CM, Esselstyn JA, Good JM, Moussalli A, Rowe KC. Molecular evolution of ecological specialisation: genomic insights from the diversification of murine rodents. Genome Biol Evol. 2021:13(7):evab103. 10.1093/gbe/evab103. 33988699 PMC8258016

[evaf017-B117] Roycroft EJ, Moussalli A, Rowe KC. Phylogenomics uncovers confidence and conflict in the rapid radiation of Australo-Papuan rodents. Syst Biol. 2020:69(3):431–444. 10.1093/sysbio/syz044. 31225616

[evaf017-B118] Sarver BA, Keeble S, Cosart T, Tucker PK, Dean MD, Good JM. Phylogenomic insights into mouse evolution using a pseudoreference approach. Genome Biol Evol. 2017:9(3):726–739. 10.1093/gbe/evx034. 28338821 PMC5381554

[evaf017-B119] Scally A, Dutheil JY, Hillier LW, Jordan GE, Goodhead I, Herrero J, Hobolth A, Lappalainen T, Mailund T, Marques-Bonet T, et al Insights into hominid evolution from the gorilla genome sequence. Nature. 2012:483(7388):169–175. 10.1038/nature10842. 22398555 PMC3303130

[evaf017-B120] Schenk JJ, Rowe KC, Steppan SJ. Ecological opportunity and incumbency in the diversification of repeated continental colonizations by muroid rodents. Syst Biol. 2013:62(6):837–864. 10.1093/sysbio/syt050. 23925508

[evaf017-B121] Schliep KP . Phangorn: phylogenetic analysis in R. Bioinformatics. 2011:27(4):592–593. 10.1093/bioinformatics/btq706. 21169378 PMC3035803

[evaf017-B122] Serizawa K, Suzuki H, Tsuchiya K. A phylogenetic view on species radiation in Apodemus inferred from variation of nuclear and mitochondrial genes. Biochem Genet. 2000:38(1/2):27–40. 10.1023/A:1001828203201. 10862357

[evaf017-B123] Shifman S, Bell JT, Copley RR, Taylor MS, Williams RW, Mott R, Flint J. A high-resolution single nucleotide polymorphism genetic map of the mouse genome. PLoS Biol. 2006:4(12):e395. 10.1371/journal.pbio.0040395. 17105354 PMC1635748

[evaf017-B124] Singhal S, Leffler EM, Sannareddy K, Turner I, Venn O, Hooper DM, Strand AI, Li Q, Raney B, Balakrishnan CN, et al Stable recombination hotspots in birds. Science. 2015:350(6263):928–932. 10.1126/science.aad0843. 26586757 PMC4864528

[evaf017-B125] Slatkin M, Pollack JL. The concordance of gene trees and species trees at two linked loci. Genetics. 2006:172(3):1979–1984. 10.1534/genetics.105.049593. 16361238 PMC1456312

[evaf017-B126] Smagulova F, Gregoretti IV, Brick K, Khil P, Camerini-Otero RD, Petukhova GV. Genome-wide analysis reveals novel molecular features of mouse recombination hotspots. Nature. 2011:472(7343):375–378. 10.1038/nature09869. 21460839 PMC3117304

[evaf017-B127] Smit AFA, Hubley R, Green P. 2013–2015. RepeatMasker Open-4.0. 4.0.

[evaf017-B128] Smith BT, Merwin J, Provost KL, Thom G, Brumfield RT, Ferreira M, Mauck WM, Moyle RG, Wright TF, Joseph L. Phylogenomic analysis of the parrots of the world distinguishes artifactual from biological sources of gene tree discordance. Syst Biol. 2023:72(1):228–241. 10.1093/sysbio/syac055. 35916751

[evaf017-B129] Smith MD, Wertheim JO, Weaver S, Murrell B, Scheffler K, Kosakovsky Pond SL. Less is more: an adaptive branch-site random effects model for efficient detection of episodic diversifying selection. Mol Biol Evol. 2015:32(5):1342–1353. 10.1093/molbev/msv022. 25697341 PMC4408413

[evaf017-B130] Smith SA, Brown JW, Walker JF. So many genes, so little time: a practical approach to divergence-time estimation in the genomic era. PLoS One. 2018:13(5):e0197433. 10.1371/journal.pone.0197433. 29772020 PMC5957400

[evaf017-B131] Smith SA, Moore MJ, Brown JW, Yang Y. Analysis of phylogenomic datasets reveals conflict, concordance, and gene duplications with examples from animals and plants. BMC Evol Biol. 2015:15(1):150. 10.1186/s12862-015-0423-0. 26239519 PMC4524127

[evaf017-B132] Stanyon R, Yang F, Cavagna P, O'Brien P, Bagga M, Ferguson-Smith M, Wienberg J. Animal cytogenetics and comparative mapping-reciprocal chromosome painting shows that genomic rearrangement between rat and mouse proceeds ten times faster than between humans and cats. Cytogenet Cell Genet. 1999:84(3-4):150–155. 10.1159/000015244. 10393417

[evaf017-B133] Stapley J, Feulner PGD, Johnston SE, Santure AW, Smadja CM. Variation in recombination frequency and distribution across eukaryotes: patterns and processes. Philos Trans R Soc Lond B Biol Sci. 2017:372(1736):20160455. 10.1098/rstb.2016.0455. 29109219 PMC5698618

[evaf017-B134] Steenwyk JL, Li Y, Zhou X, Shen XX, Rokas A. Incongruence in the phylogenomics era. Nat Rev Genet. 2023:24(12):834–850. 10.1038/s41576-023-00620-x. 37369847 PMC11499941

[evaf017-B135] Steppan SJ, Adkins RM, Spinks PQ, Hale C. Multigene phylogeny of the Old World mice, Murinae, reveals distinct geographic lineages and the declining utility of mitochondrial genes compared to nuclear genes. Mol Phylogenet Evol. 2005:37(2):370–388. 10.1016/j.ympev.2005.04.016. 15975830

[evaf017-B136] Steppan SJ, Schenk JJ. Muroid rodent phylogenetics: 900-species tree reveals increasing diversification rates. PLoS One. 2017:12(8):e0183070. 10.1371/journal.pone.0183070. 28813483 PMC5559066

[evaf017-B137] Stevison LS, Woerner AE, Kidd JM, Kelley JL, Veeramah KR, McManus KF, Great Ape Genome Project; Bustamante CD, Hammer MF, Wall JD. The time scale of recombination rate evolution in great apes. Mol Biol Evol. 2016:33(4):928–945. 10.1093/molbev/msv331. 26671457 PMC5870646

[evaf017-B138] Sun C, Huang J, Wang Y, Zhao X, Su L, Thomas GWC, Zhao M, Zhang X, Jungreis I, Kellis M, et al Genus-wide characterization of bumblebee genomes provides insights into their evolution and variation in ecological and behavioral traits. Mol Biol Evol. 2021:38(2):486–501. 10.1093/molbev/msaa240. 32946576 PMC7826183

[evaf017-B139] Suzuki H, Shimada T, Terashima M, Tsuchiya K, Aplin K. Temporal, spatial, and ecological modes of evolution of Eurasian Mus based on mitochondrial and nuclear gene sequences. Mol Phylogenet Evol. 2004:33(3):626–646. 10.1016/j.ympev.2004.08.003. 15522792

[evaf017-B140] Tange O . 2018. GNU Parallel. 20240322. 10.5281/zenodo.14550073.

[evaf017-B141] To TH, Jung M, Lycett S, Gascuel O. Fast dating using least-squares criteria and algorithms. Syst Biol. 2016:65(1):82–97. 10.1093/sysbio/syv068. 26424727 PMC4678253

[evaf017-B142] Treaster S, Deelen J, Daane JM, Murabito J, Karasik D, Harris MP. Convergent genomics of longevity in rockfishes highlights the genetics of human life span variation. Sci Adv. 2023:9(2):eadd2743. 10.1126/sciadv.add2743. 36630509 PMC9833670

[evaf017-B143] Vanderpool D, Minh BQ, Lanfear R, Hughes D, Murali S, Harris RA, Raveendran M, Muzny DM, Hibbins MS, Williamson RJ, et al Primate phylogenomics uncovers multiple rapid radiations and ancient interspecific introgression. PLoS Biol. 2020:18(12):e3000954. 10.1371/journal.pbio.3000954. 33270638 PMC7738166

[evaf017-B144] van der Valk T, Pecnerova P, Diez-Del-Molino D, Bergstrom A, Oppenheimer J, Hartmann S, Xenikoudakis G, Thomas JA, Dehasque M, Saglican E, et al Million-year-old DNA sheds light on the genomic history of mammoths. Nature. 2021:591(7849):265–269. 10.1038/s41586-021-03224-9. 33597750 PMC7116897

[evaf017-B145] Wang LG, Lam TT, Xu S, Dai Z, Zhou L, Feng T, Guo P, Dunn CW, Jones BR, Bradley T, et al Treeio: an R package for phylogenetic tree input and output with richly annotated and associated data. Mol Biol Evol. 2020:37(2):599–603. 10.1093/molbev/msz240. 31633786 PMC6993851

[evaf017-B146] Mouse Genome Sequencing Consortium; Waterston RH, Lindblad-Toh K, Birney E, Rogers J, Abril JF, Agarwal P, Agarwala R, Ainscough R, Alexandersson M, et al Initial sequencing and comparative analysis of the mouse genome. Nature. 2002:420(6915):520–562. 10.1038/nature01262. 12466850

[evaf017-B147] White MA, Ane C, Dewey CN, Larget BR, Payseur BA. Fine-scale phylogenetic discordance across the house mouse genome. PLoS Genet. 2009:5(11):e1000729. 10.1371/journal.pgen.1000729. 19936022 PMC2770633

[evaf017-B148] Yalcin B, Wong K, Agam A, Goodson M, Keane TM, Gan X, Nellaker C, Goodstadt L, Nicod J, Bhomra A, et al Sequence-based characterization of structural variation in the mouse genome. Nature. 2011:477(7364):326–329. 10.1038/nature10432. 21921916 PMC3428933

[evaf017-B149] Yang Z . PAML 4: phylogenetic analysis by maximum likelihood. Mol Biol Evol. 2007:24(8):1586–1591. 10.1093/molbev/msm088. 17483113

[evaf017-B150] Yekutieli D, Benjamini Y. Resampling-based false discovery rate controlling multiple test procedures for correlated test statistics. J Stat Plan Inference. 1999:82(1-2):171–196. 10.1016/S0378-3758(99)00041-5.

[evaf017-B151] Yu G . Using ggtree to visualize data on tree-like structures. Curr Protoc Bioinformatics. 2020:69(1):e96. 10.1002/cpbi.96. 32162851

[evaf017-B152] Yu G, Smith DK, Zhu H, Guan Y, Lam TT-Y. Ggtree: an r package for visualization and annotation of phylogenetic trees with their covariates and other associated data. Methods Ecol Evol. 2017:8(1):28–36. 10.1111/2041-210X.12628.

[evaf017-B153] Zhang C, Rabiee M, Sayyari E, Mirarab S. ASTRAL-III: polynomial time species tree reconstruction from partially resolved gene trees. BMC Bioinformatics. 2018:19(S6):153. 10.1186/s12859-018-2129-y. 29745866 PMC5998893

