# Supplementary material for: The Genomic Landscape, Causes, and Consequences of Extensive Phylogenomic Discordance in Murine Rodents
Source: Genome Biol Evol. 2025 Feb 4;17(2):evaf017. doi: 10.1093/gbe/evaf017 (PMC11837218; doi:10.1093/gbe/evaf017)

# Rodent phylogenies: 10kb windows on chr1

## Chromosome length: 195471971bp, showing 12112 of 19548 windows, 184 topologies

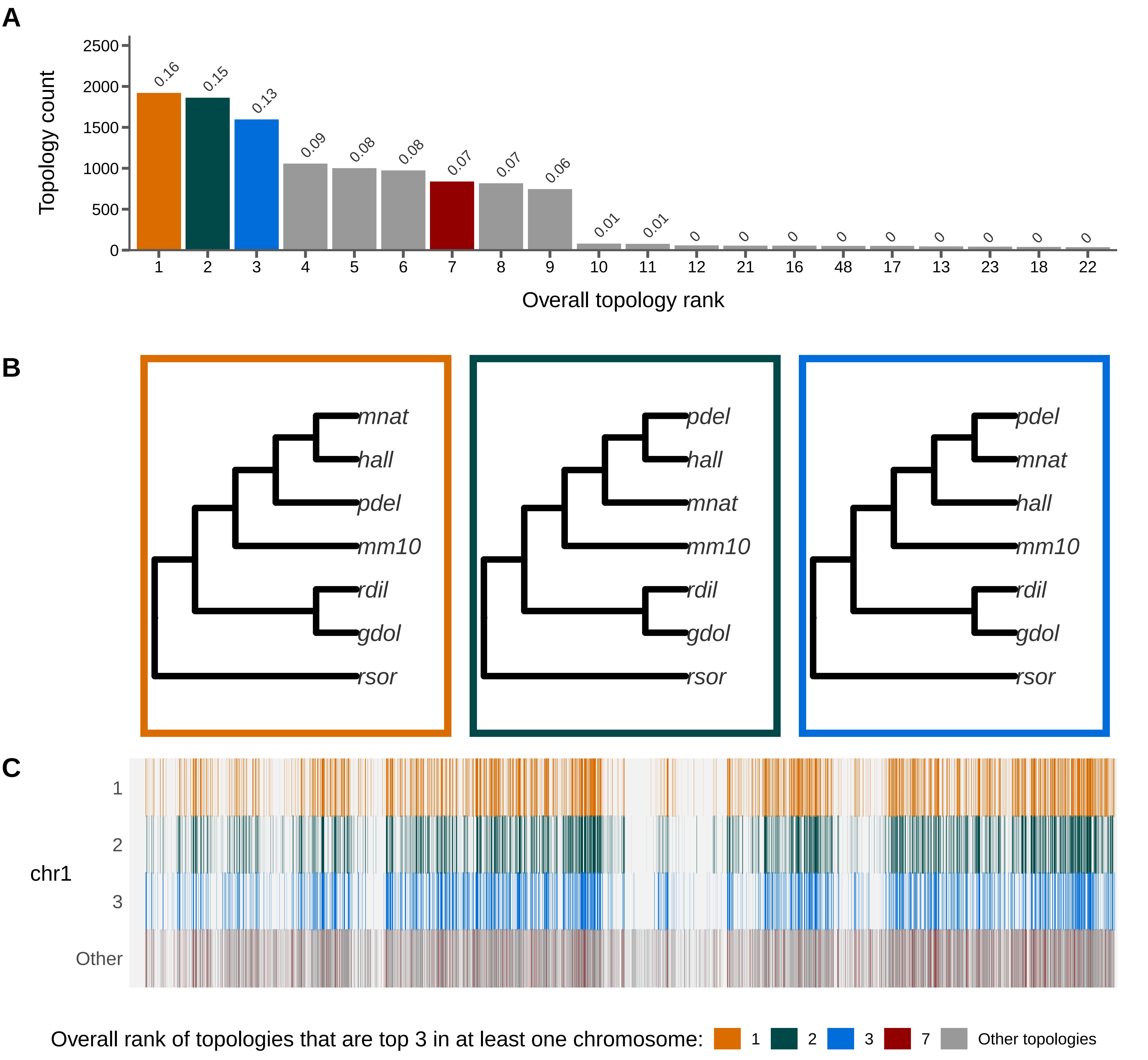

# Rodent phylogenies: 10kb windows on chr2

## Chromosome length: 182113224bp, showing 12386 of 18212 windows, 123 topologies

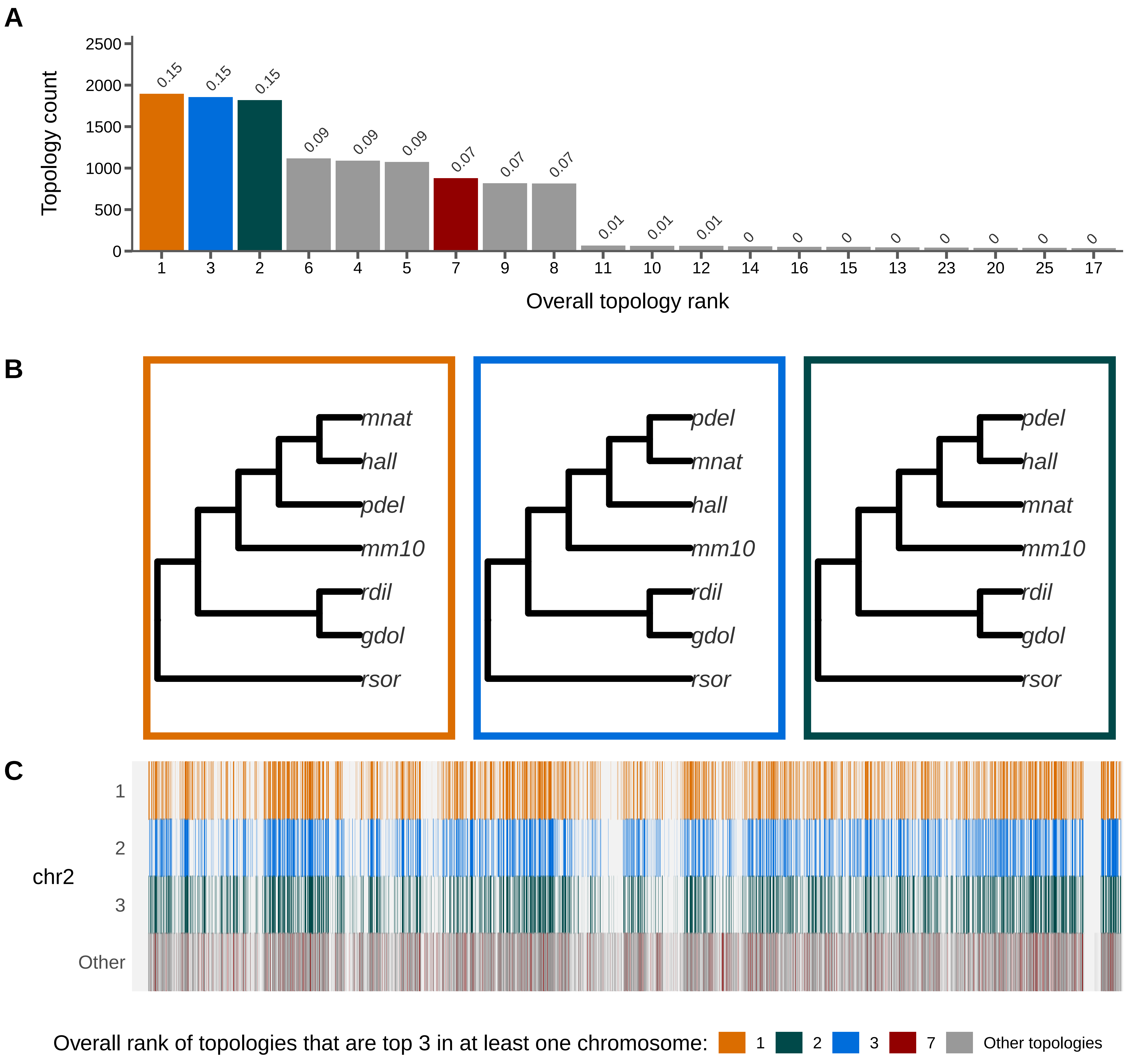

# Rodent phylogenies: 10kb windows on chr3

**Chromosome length: 160039680bp, showing 9721 of 16004 windows, 114 topologies**

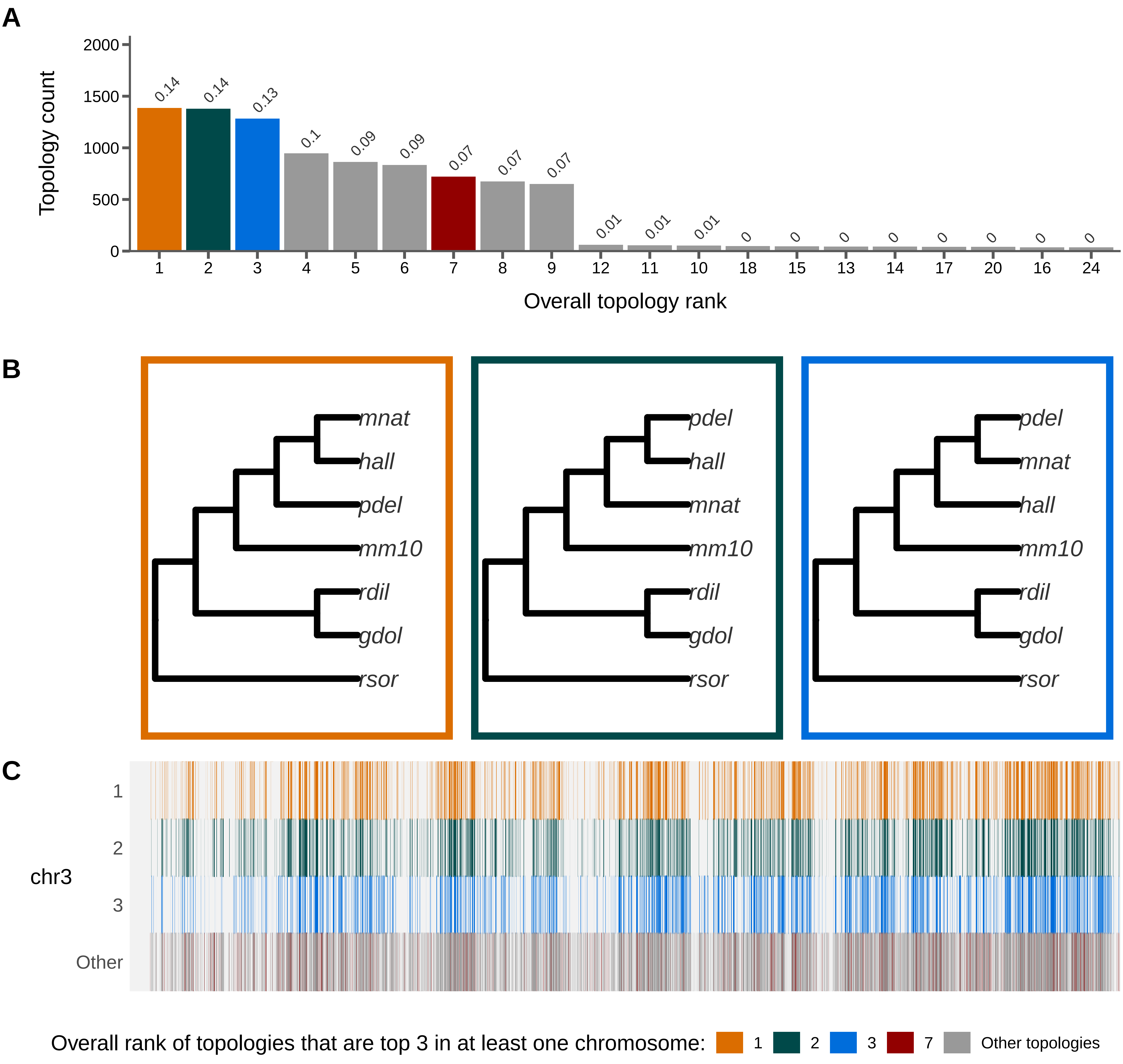

# Rodent phylogenies: 10kb windows on chr4

**Chromosome length: 156508116bp, showing 9363 of 15651 windows, 144 topologies**

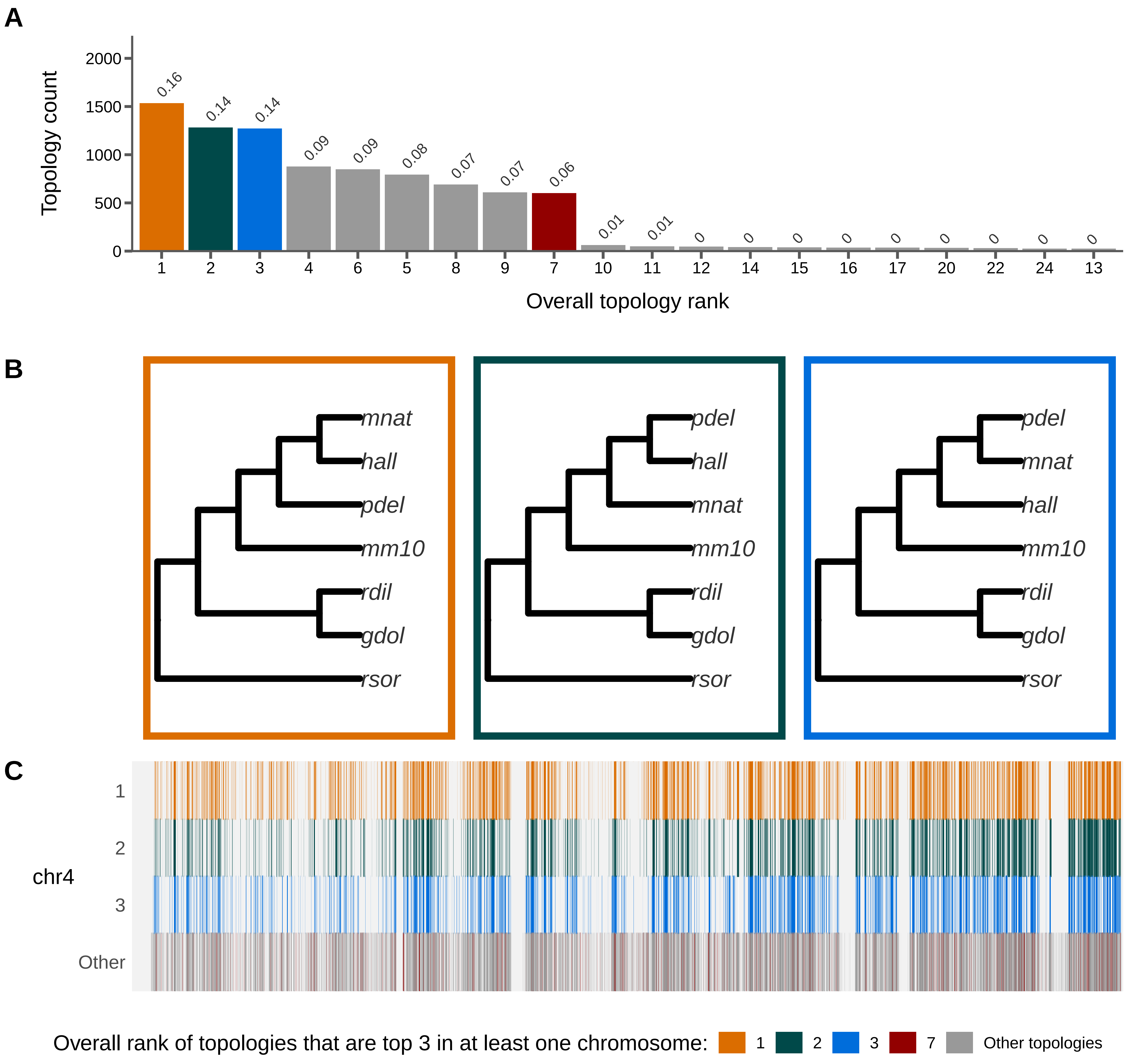

# Rodent phylogenies: 10kb windows on chr5

**Chromosome length: 151834684bp, showing 9859 of 15184 windows, 134 topologies**

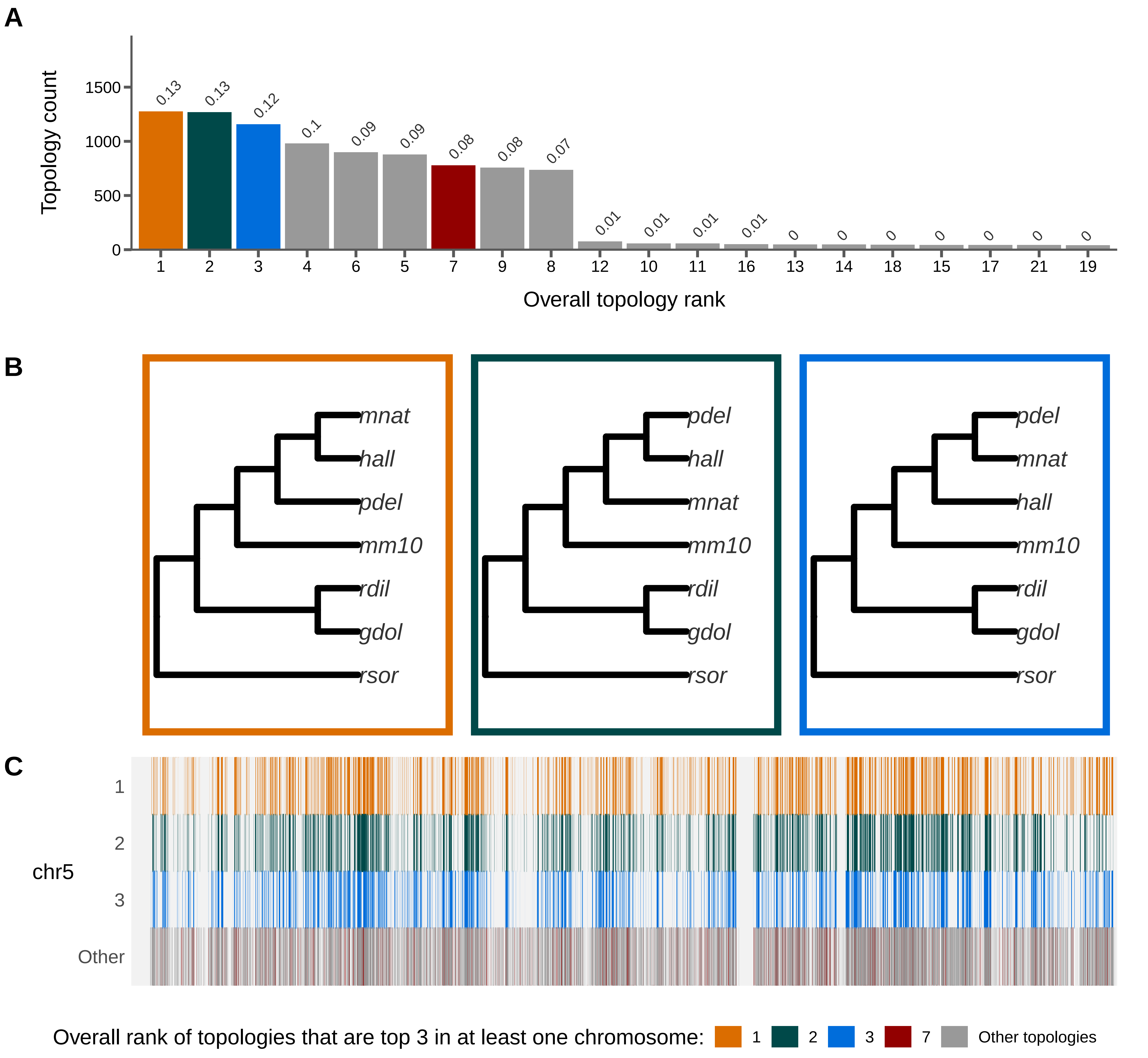

# Rodent phylogenies: 10kb windows on chr6

## Chromosome length: 149736546bp, showing 9563 of 14974 windows, 172 topologies

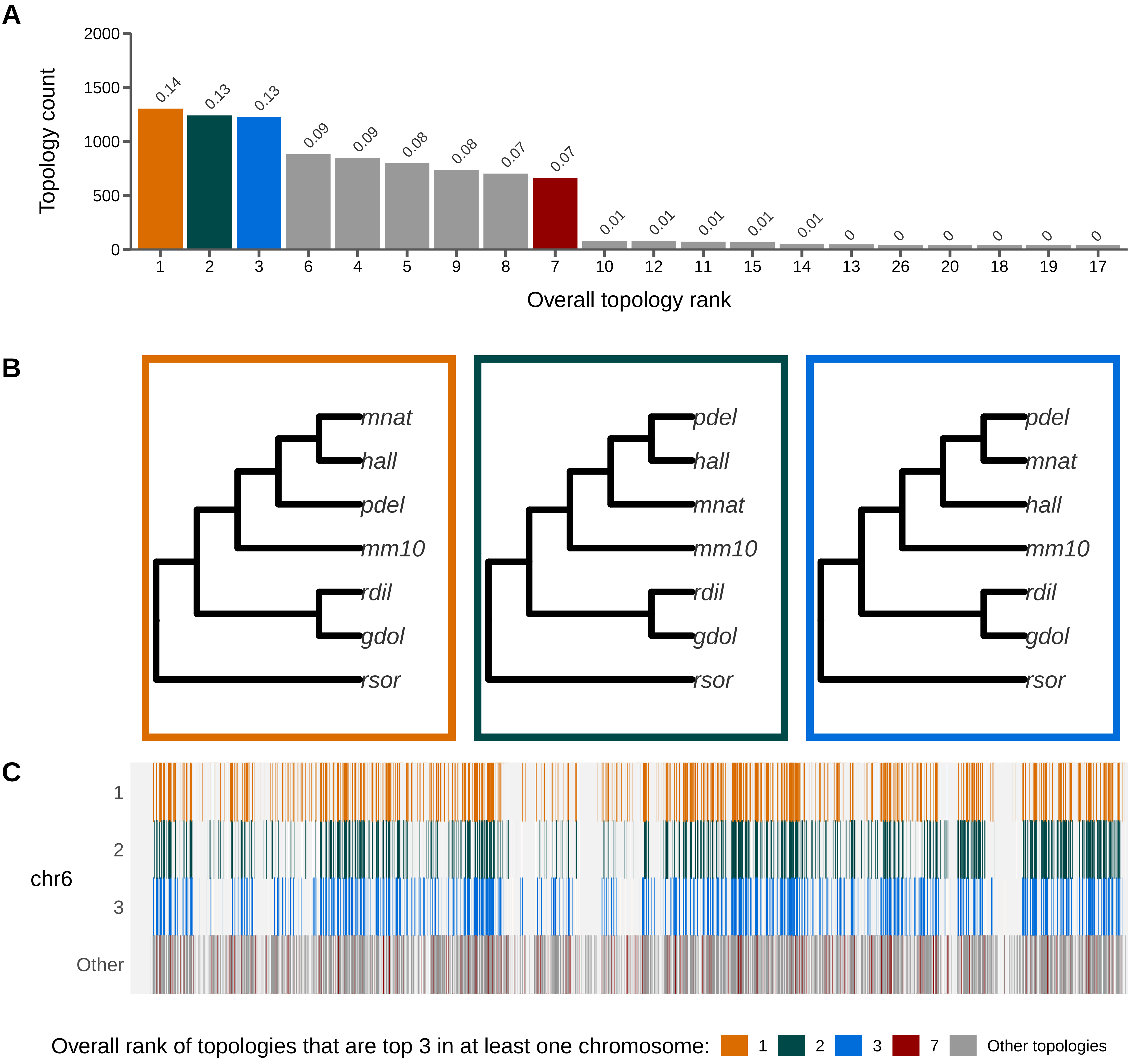

# Rodent phylogenies: 10kb windows on chr7

**Chromosome length: 145441459bp, showing 8387 of 14545 windows, 218 topologies**

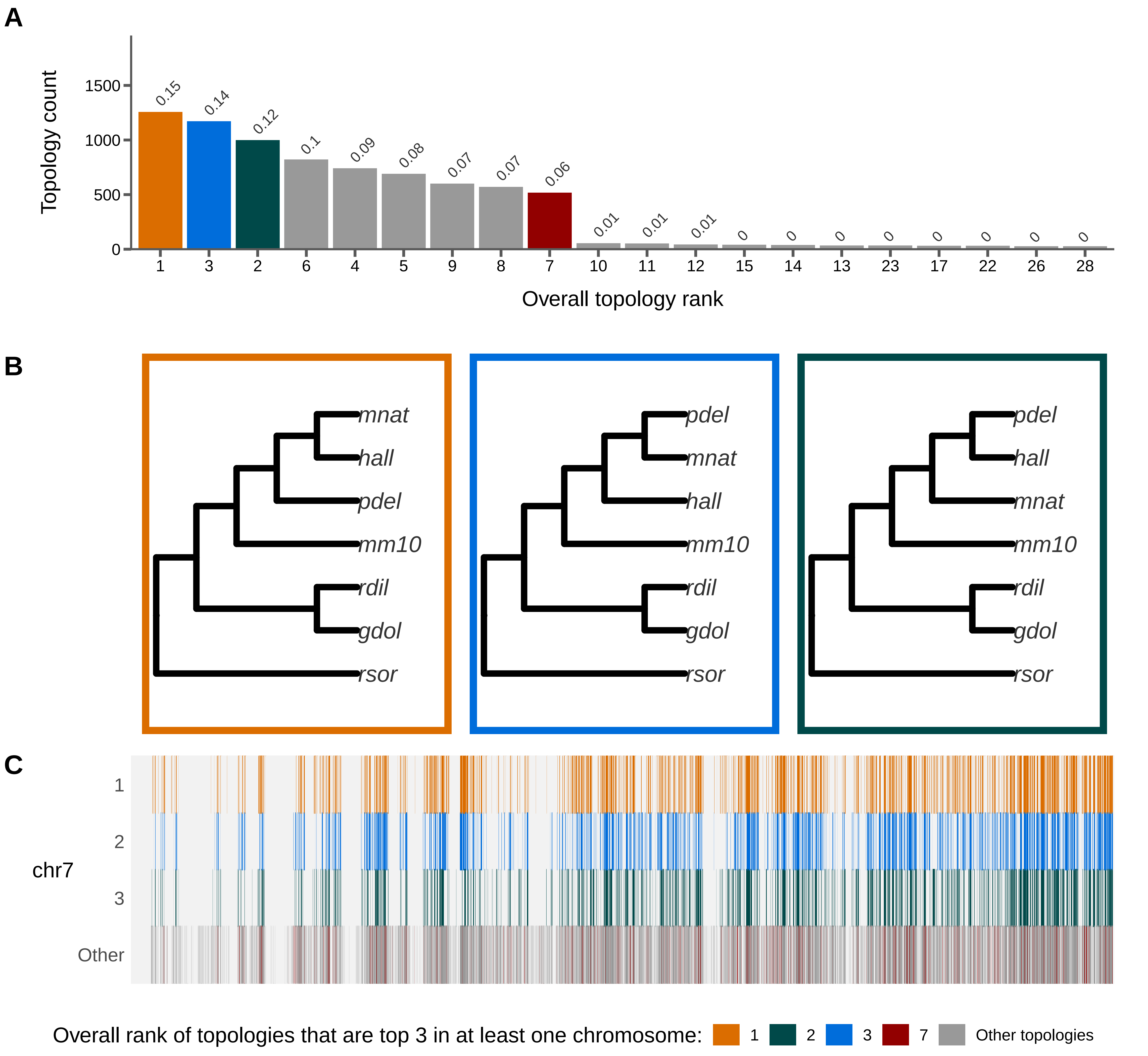

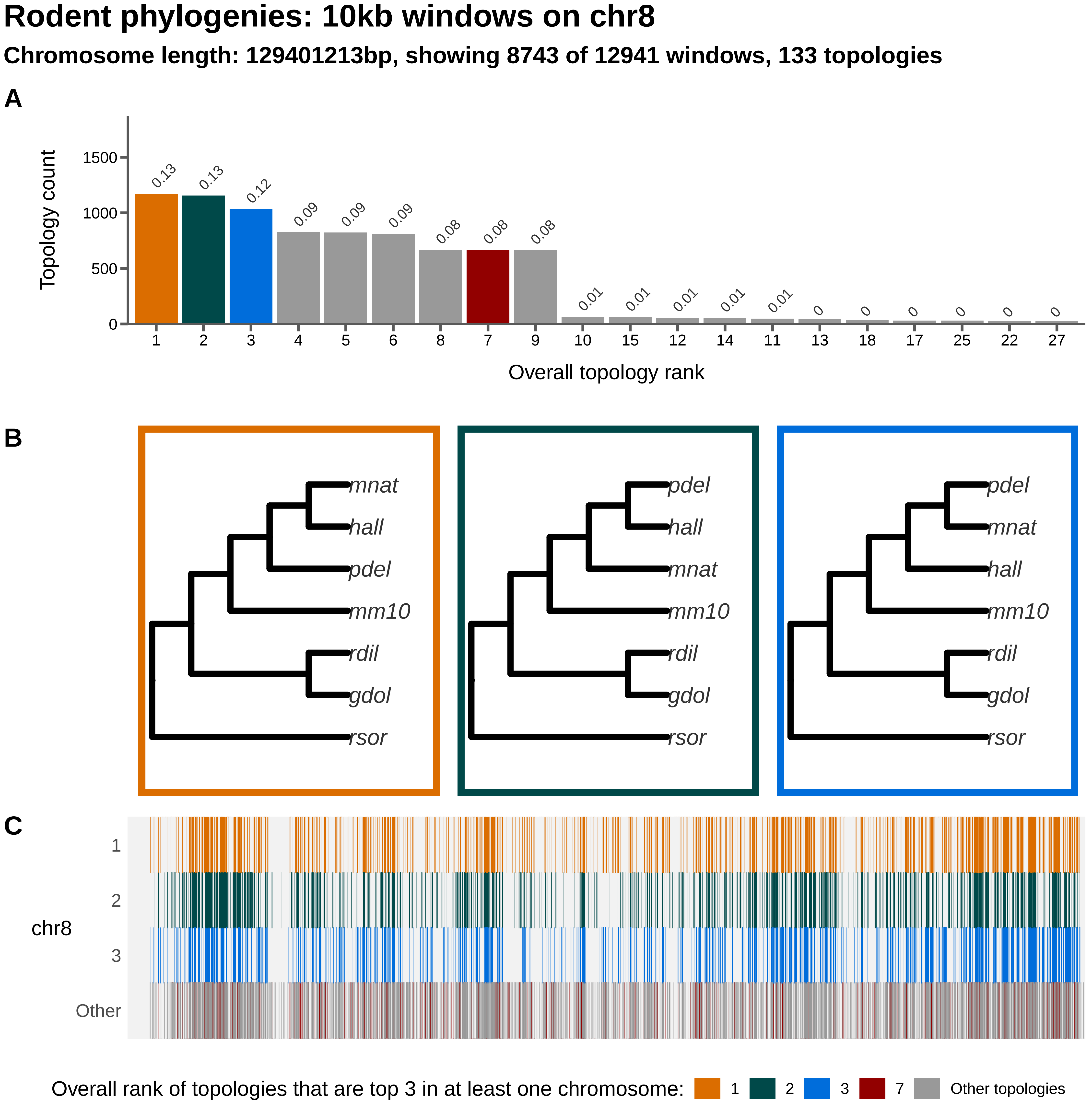
Rodent phylogenies: 10kb windows on chr8
Chromosome length: 129401213bp, showing 8743 of 12941 windows, 133 topologies
A
Topology count
1500
1000
500
0
0.13
0.13
0.12
0.09
0.09
0.09
0.08
0.08
0.08
0.01
0.01
0.01
0.01
0.01
0
0
0
0
0
0
1
2
3
4
5
6
8
7
9
10
15
12
14
11
13
18
17
25
22
27
Overall topology rank
B
mnat
hall
pdel
mm10
rdil
gdol
rsor
pdel
hall
mnat
mm10
rdil
gdol
rsor
pdel
mnat
hall
mm10
rdil
gdol
rsor
C
chr8
1
2
3
Other
Overall rank of topologies that are top 3 in at least one chromosome:
1
2
3
7
Other topologies

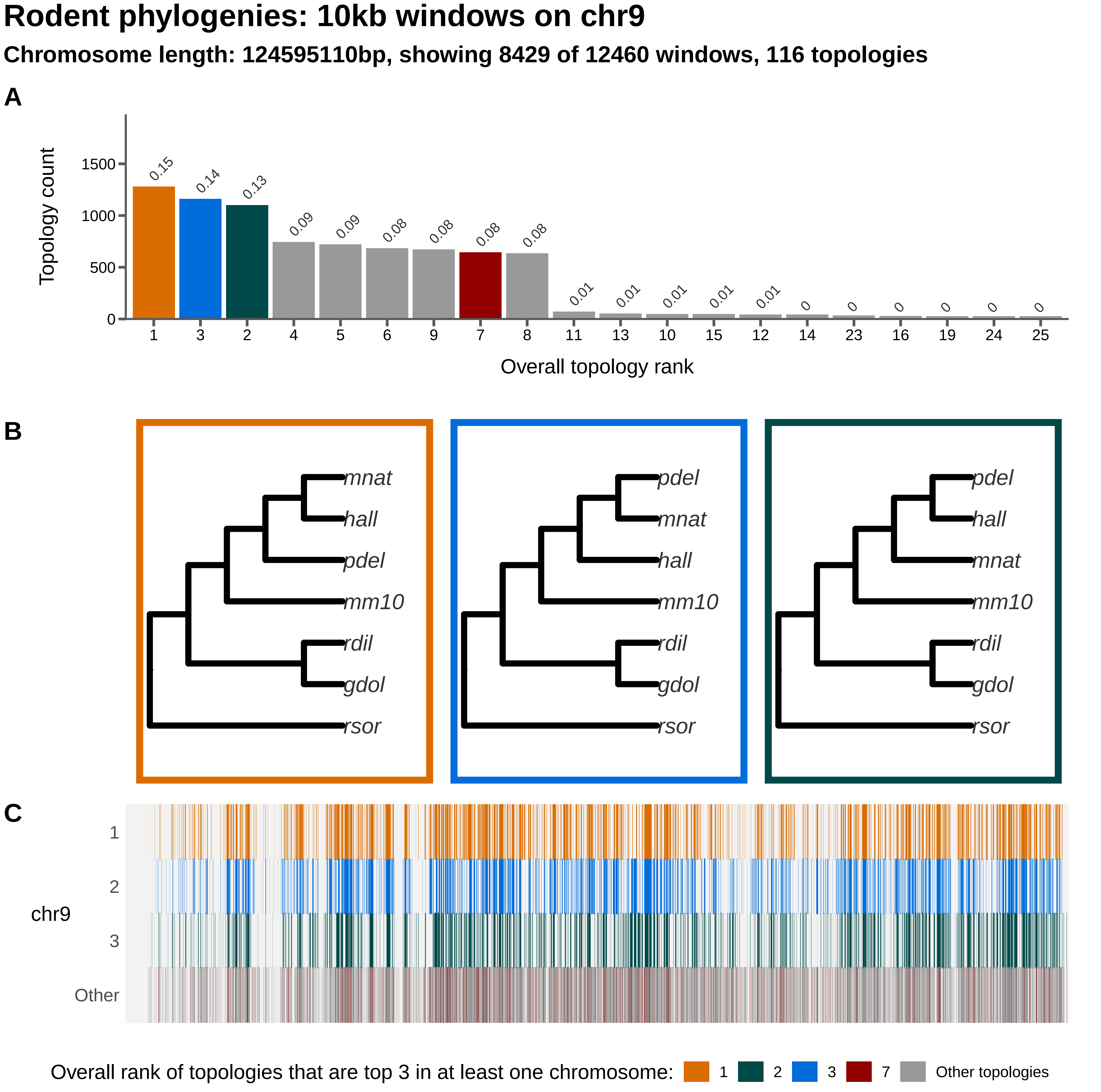
Rodent phylogenies: 10kb windows on chr9
Chromosome length: 124595110bp, showing 8429 of 12460 windows, 116 topologies
A
Topology count
1500
1000
500
0
0.15
0.14
0.13
0.09
0.09
0.08
0.08
0.08
0.08
0.01
0.01
0.01
0.01
0.01
0
0
0
0
0
0
1
3
2
4
5
6
9
7
8
11
13
10
15
12
14
23
16
19
24
25
Overall topology rank
B
mnat
hall
pdel
mm10
rdil
gdol
rsor
pdel
mnat
hall
mm10
rdil
gdol
rsor
pdel
hall
mnat
mm10
rdil
gdol
rsor
C
1
2
chr9
3
Other
Overall rank of topologies that are top 3 in at least one chromosome:
1
2
3
7
Other topologies

# Rodent phylogenies: 10kb windows on chr10

**Chromosome length: 130694993bp, showing 8506 of 13070 windows, 110 topologies**

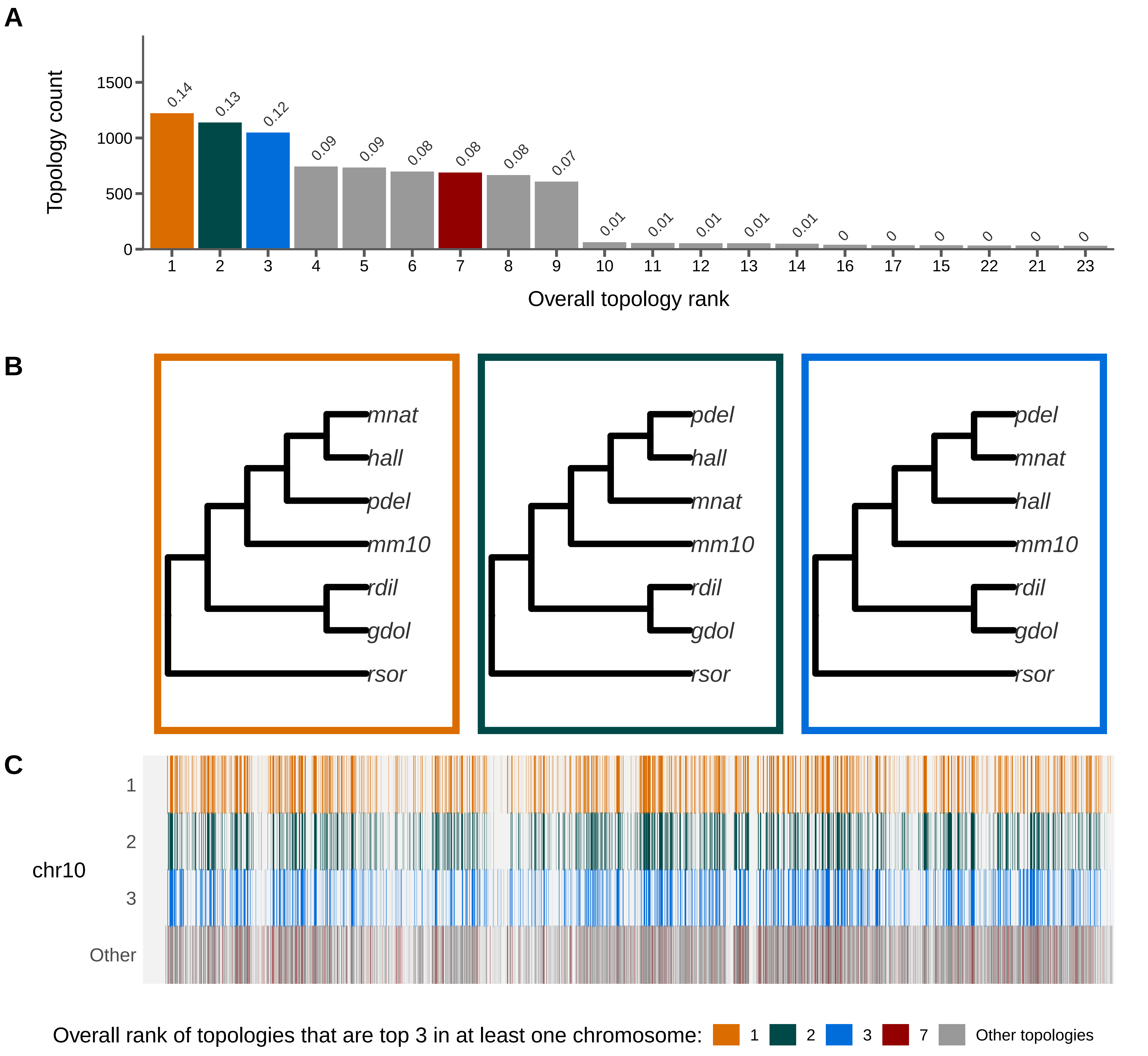

# Rodent phylogenies: 10kb windows on chr11

**Chromosome length: 122082543bp, showing 8691 of 12209 windows, 93 topologies**

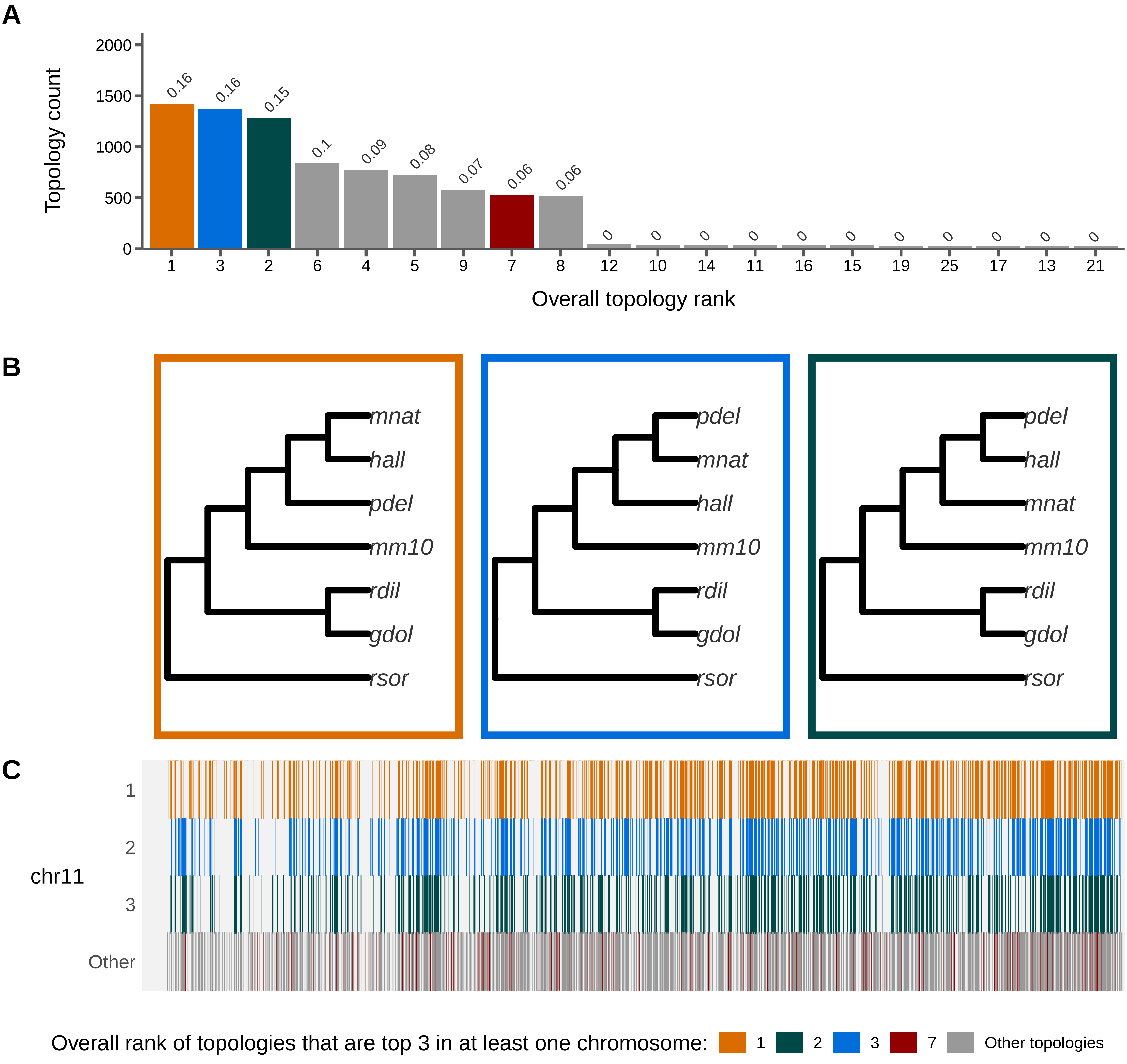

# Rodent phylogenies: 10kb windows on chr12

## Chromosome length: 120129022bp, showing 7630 of 12013 windows, 179 topologies

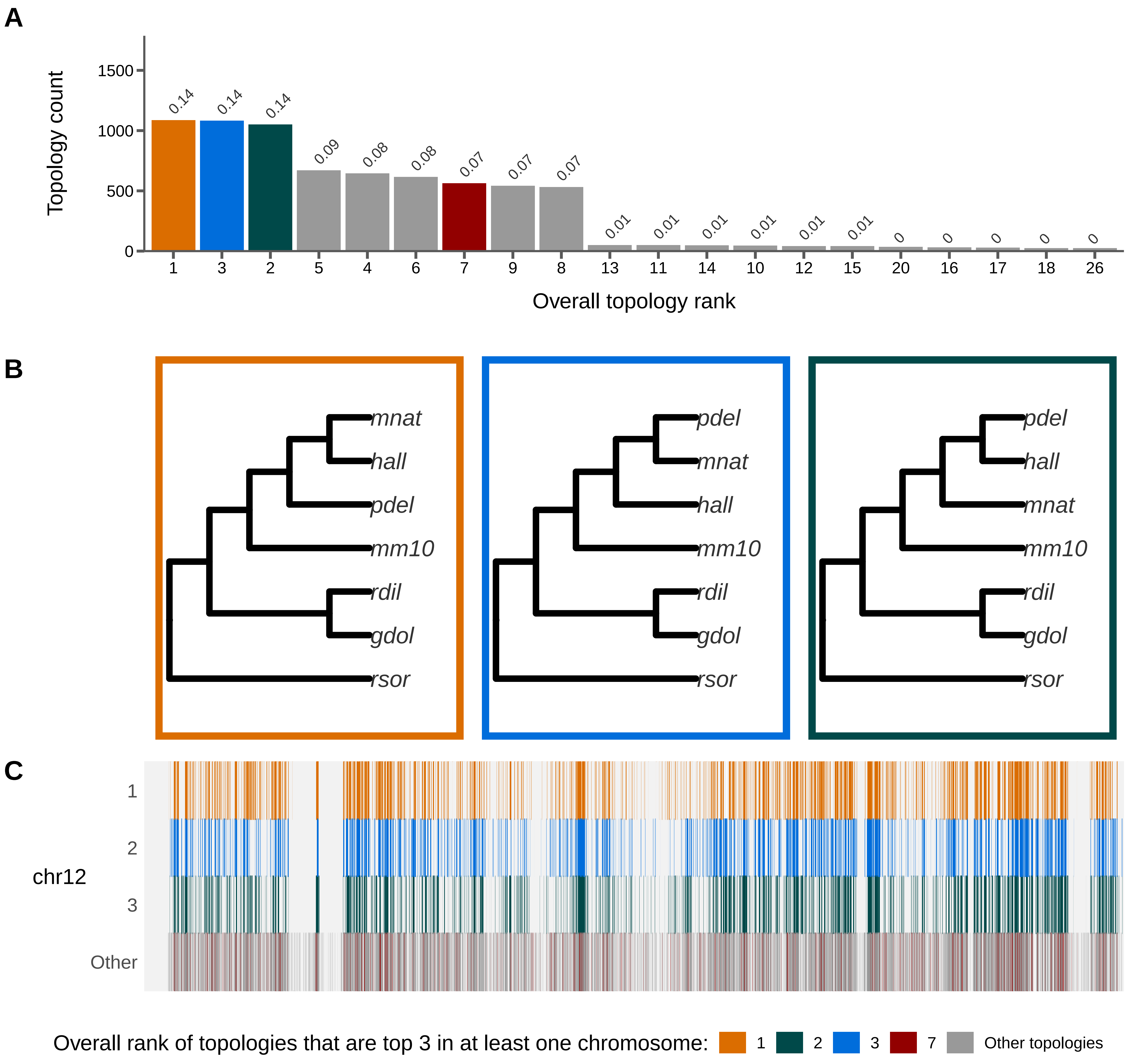

# Rodent phylogenies: 10kb windows on chr13

## Chromosome length: 120421639bp, showing 7749 of 12043 windows, 186 topologies

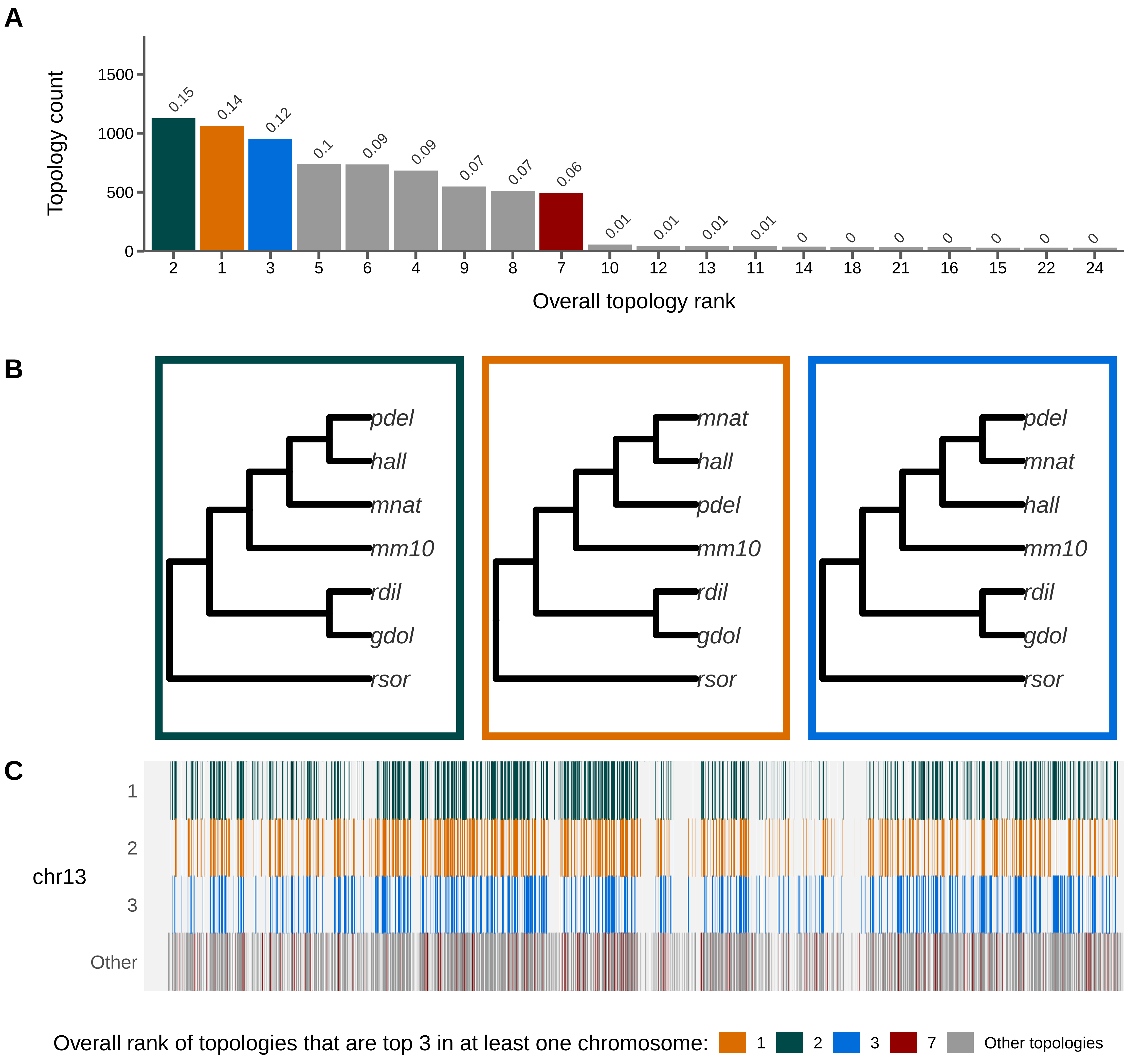

# Rodent phylogenies: 10kb windows on chr14

## Chromosome length: 124902244bp, showing 7573 of 12491 windows, 173 topologies

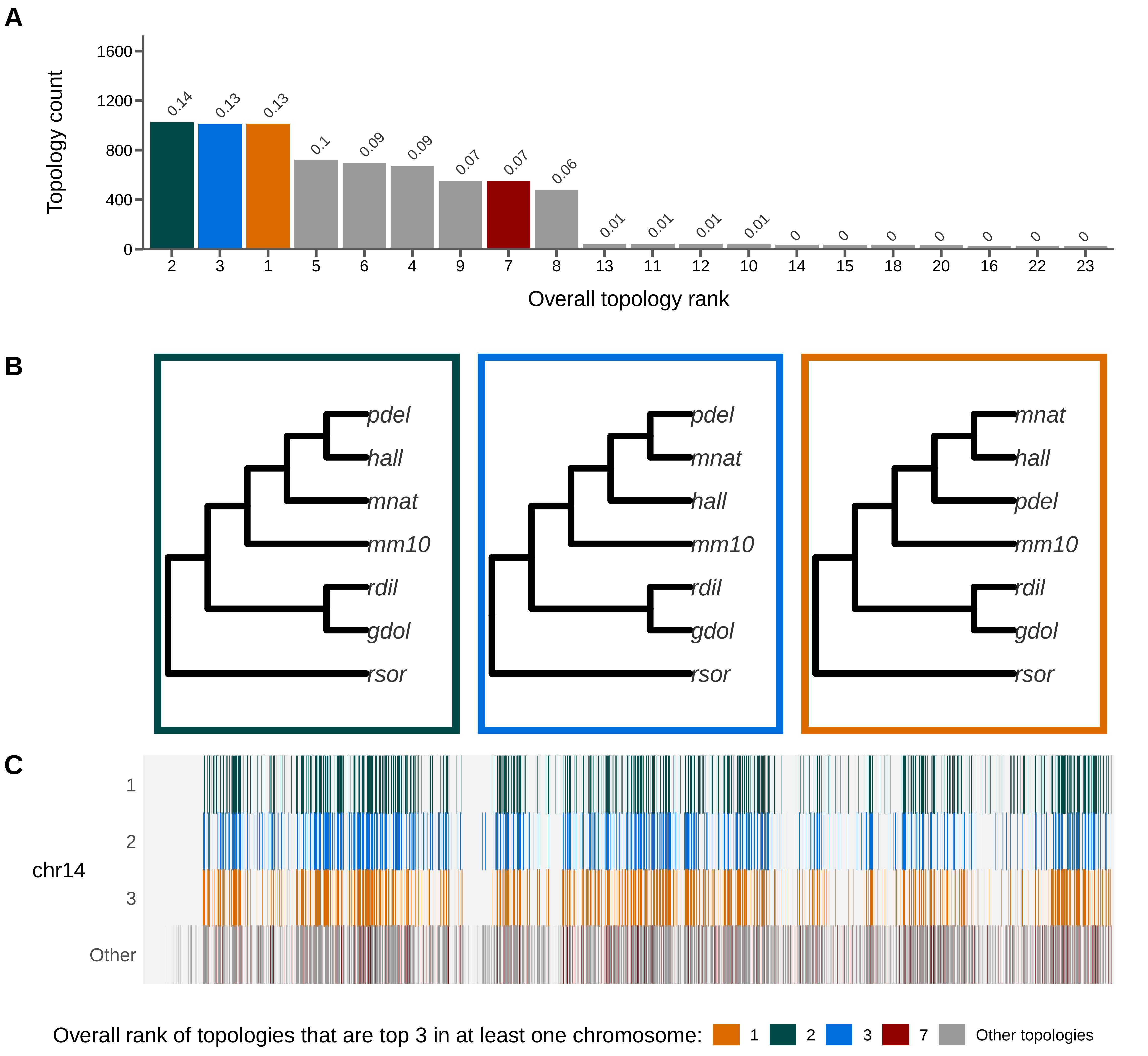

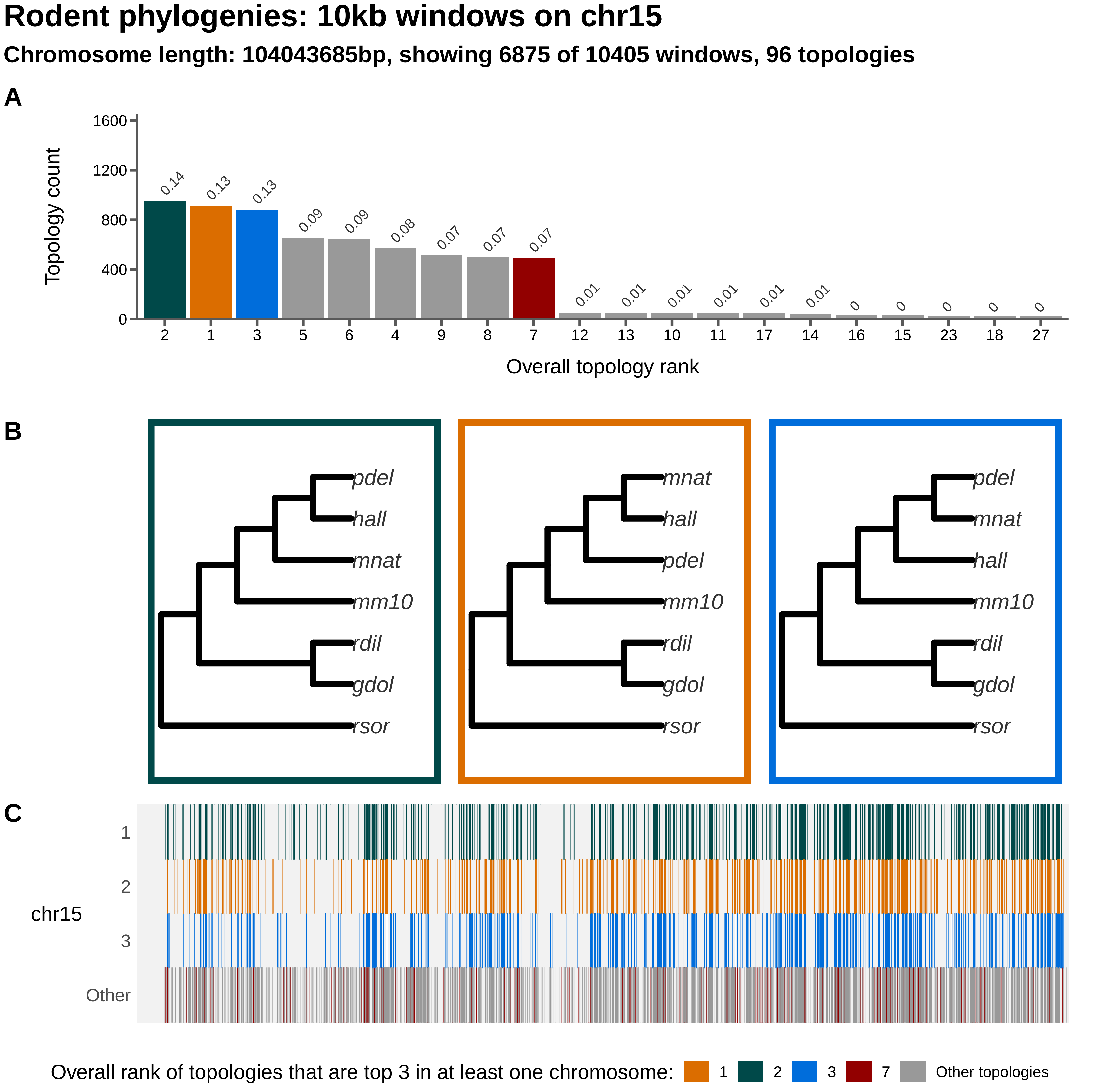
Rodent phylogenies: 10kb windows on chr15
Chromosome length: 104043685bp, showing 6875 of 10405 windows, 96 topologies
A
Topology count
1600
1200
800
400
0
0.14
0.13
0.13
0.09
0.09
0.08
0.07
0.07
0.07
0.01
0.01
0.01
0.01
0.01
0.01
0
0
0
0
0
2
1
3
5
6
4
9
8
7
12
13
10
11
17
14
16
15
23
18
27
Overall topology rank
B
pdel
hall
mnat
mm10
rdil
gdol
rsor
mnat
hall
pdel
mm10
rdil
gdol
rsor
pdel
mnat
hall
mm10
rdil
gdol
rsor
C
chr15
1
2
3
Other
Overall rank of topologies that are top 3 in at least one chromosome:
1
2
3
7
Other topologies

# Rodent phylogenies: 10kb windows on chr16

**Chromosome length: 98207768bp, showing 6451 of 9821 windows, 94 topologies**

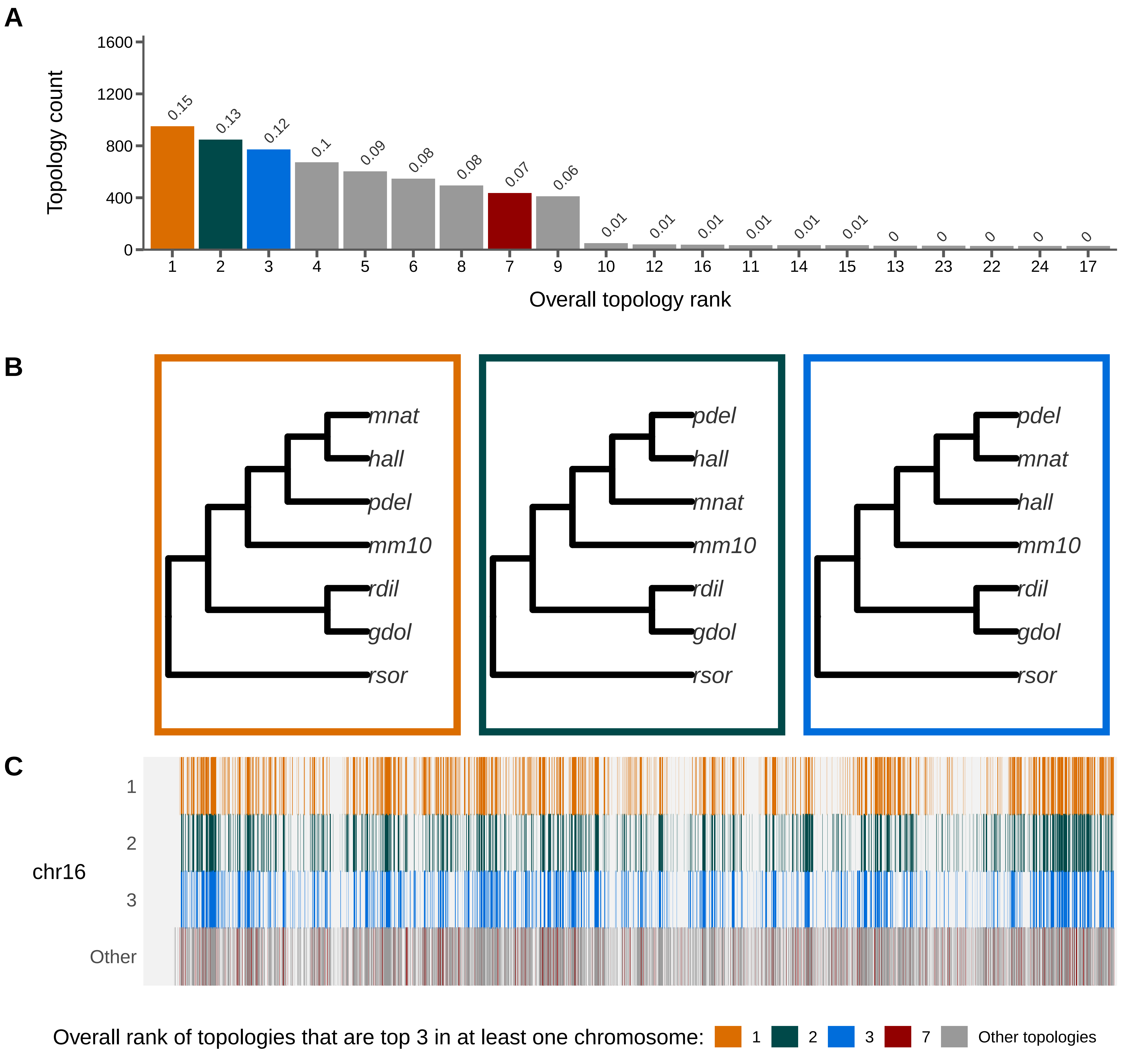

# Rodent phylogenies: 10kb windows on chr17

**Chromosome length: 94987271bp, showing 5995 of 9499 windows, 188 topologies**

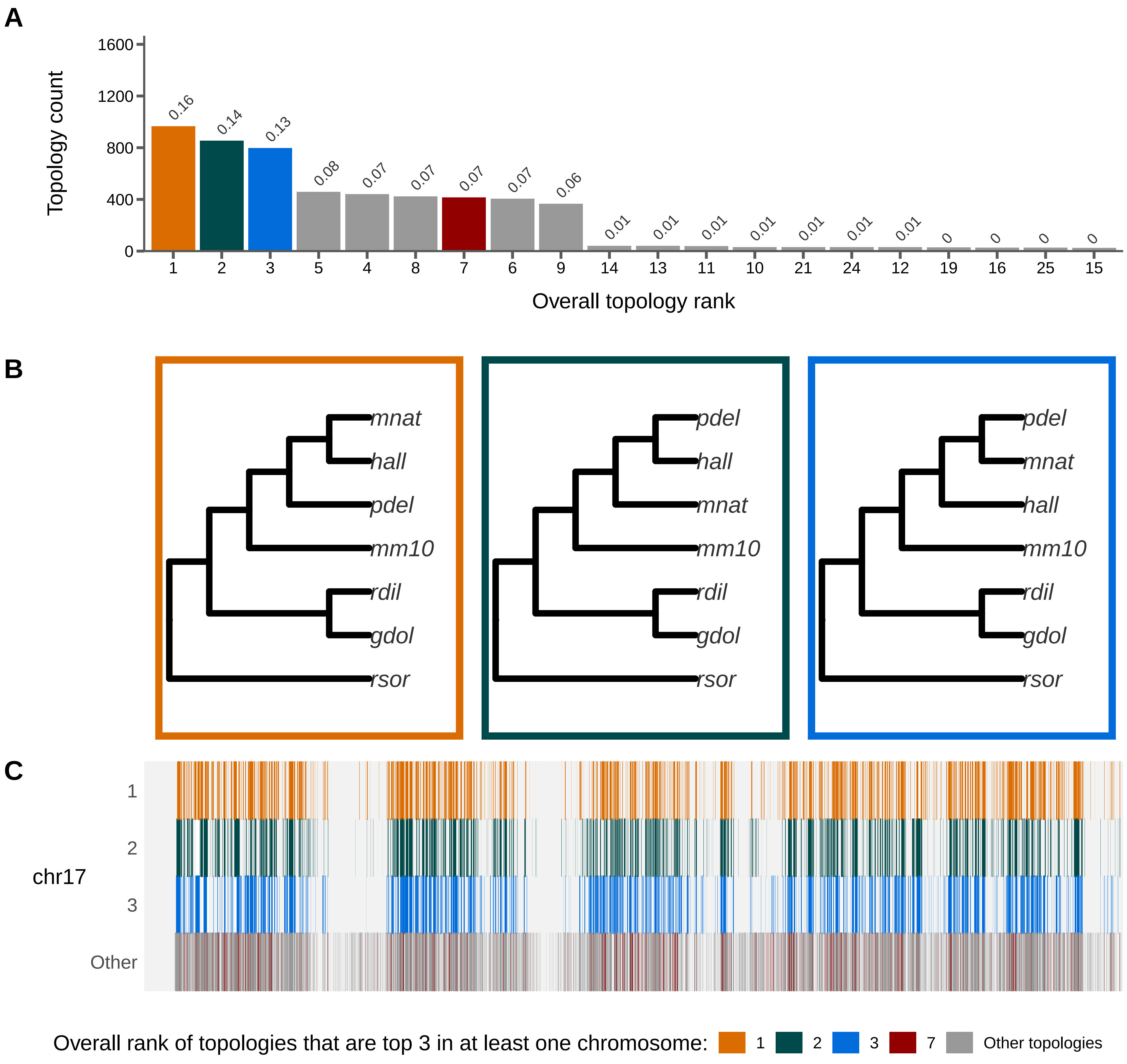

# Rodent phylogenies: 10kb windows on chr18

**Chromosome length: 90702639bp, showing 5876 of 9071 windows, 75 topologies**

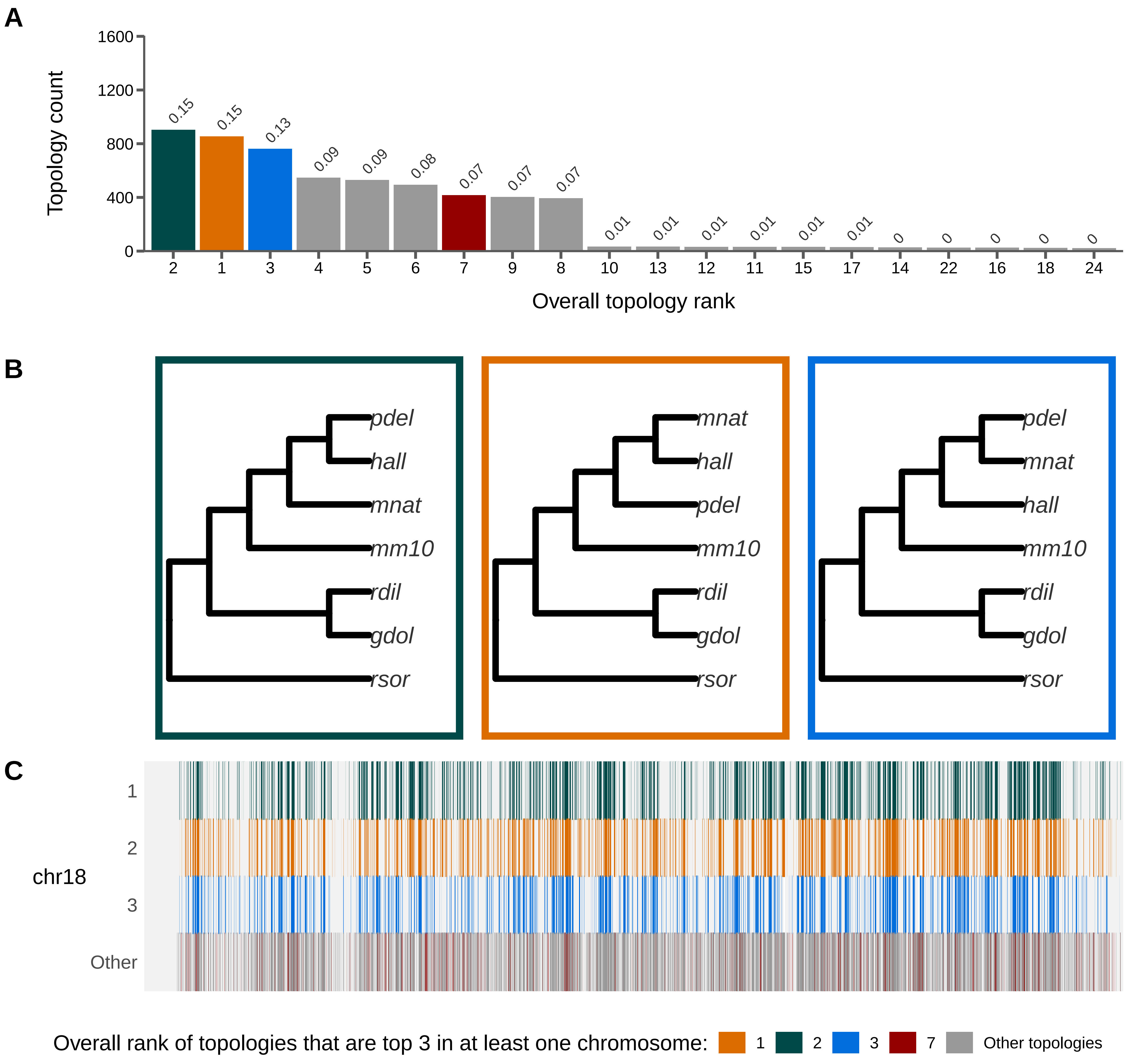

# Rodent phylogenies: 10kb windows on chr19

**Chromosome length: 61431566bp, showing 4173 of 6144 windows, 82 topologies**

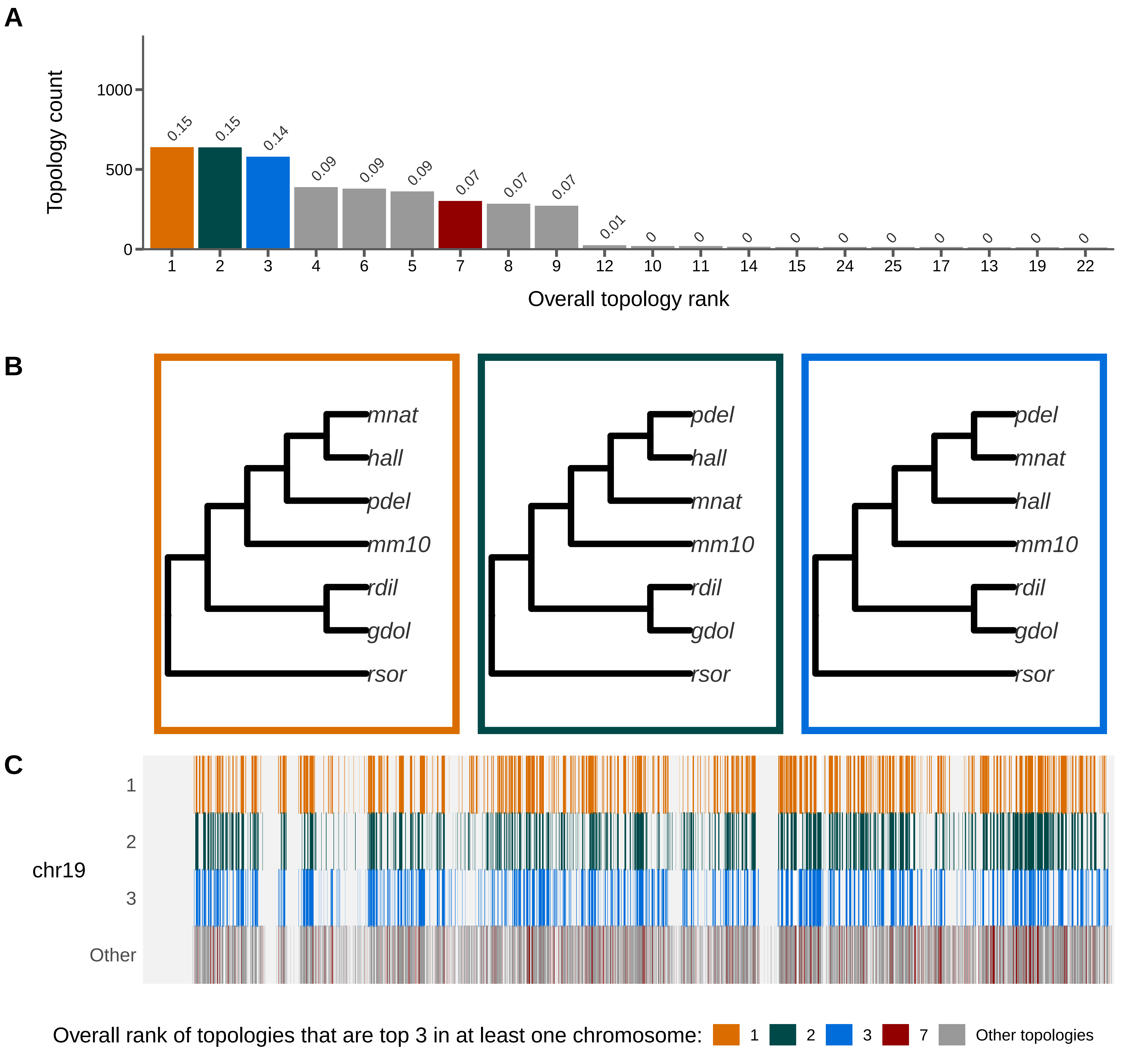

## Rodent phylogenies: 10kb windows on chrX

### Chromosome length: 171031299bp, showing 5683 of 17104 windows, 207 topologies

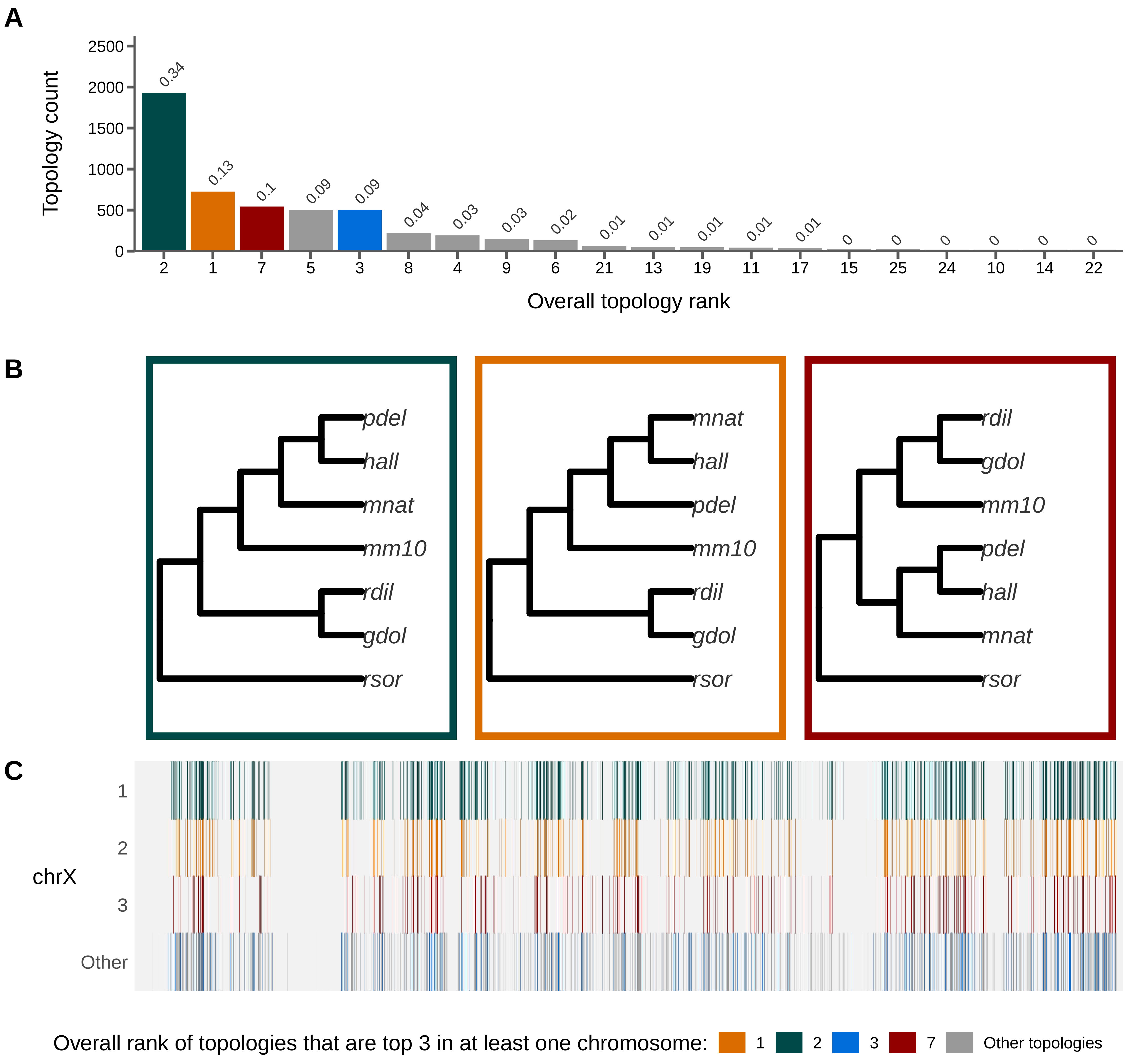

Supplement: evaf017_Supplementary_Data [file evaf017_supplementary_data.zip › fileS1.pdf]
